# Morphological and Molecular Characterization of Orchid Fruit Development

**DOI:** 10.3389/fpls.2019.00137

**Published:** 2019-02-19

**Authors:** Anita Dirks-Mulder, Israa Ahmed, Mark uit het Broek, Louie Krol, Nino Menger, Jasmijn Snier, Anne van Winzum, Anneke de Wolf, Martijn van't Wout, Jamie J. Zeegers, Roland Butôt, Reinout Heijungs, Bertie Joan van Heuven, Jaco Kruizinga, Rob Langelaan, Erik F. Smets, Wim Star, Marian Bemer, Barbara Gravendeel

**Affiliations:** ^1^Endless Forms Group, Naturalis Biodiversity Center, Leiden, Netherlands; ^2^Faculty of Science and Technology, University of Applied Sciences Leiden, Leiden, Netherlands; ^3^Department of Econometrics and Operations Research, School of Business and Economics, Vrije Universiteit Amsterdam, Amsterdam, Netherlands; ^4^Institute of Environmental Sciences (CML), Leiden University, Leiden, Netherlands; ^5^Hortus botanicus, Leiden University, Leiden, Netherlands; ^6^Institute of Biology Leiden, Leiden University, Leiden, Netherlands; ^7^Ecology, Evolution and Biodiversity Conservation Cluster, KU Leuven, Leuven, Belgium; ^8^Department of Plant Sciences, Laboratory of Molecular Biology, Wageningen, Netherlands

**Keywords:** cuticle layer, *Erycina pusilla*, fruit-gene and protein network, lignification, MADS-box genes, fruit ontogeny

## Abstract

Efficient seed dispersal in flowering plants is enabled by the development of fruits, which can be either dehiscent or indehiscent. Dehiscent fruits open at maturity to shatter the seeds, while indehiscent fruits do not open and the seeds are dispersed in various ways. The diversity in fruit morphology and seed shattering mechanisms is enormous within the flowering plants. How these different fruit types develop and which molecular networks are driving fruit diversification is still largely unknown, despite progress in eudicot model species. The orchid family, known for its astonishing floral diversity, displays a huge variation in fruit dehiscence types, which have been poorly investigated. We undertook a combined approach to understand fruit morphology and dehiscence in different orchid species to get more insight into the molecular network that underlies orchid fruit development. We describe fruit development in detail for the epiphytic orchid species *Erycina pusilla* and compare it to two terrestrial orchid species: *Cynorkis fastigiata* and *Epipactis helleborine*. Our anatomical analysis provides further evidence for the split carpel model, which explains the presence of three fertile and three sterile valves in most orchid species. Interesting differences were observed in the lignification patterns of the dehiscence zones. While *C. fastigiata* and *E. helleborine* develop a lignified layer at the valve boundaries, *E. pusilla* fruits did not lignify at these boundaries, but formed a cuticle-like layer instead. We characterized orthologs of fruit-associated MADS-domain transcription factors and of the Arabidopsis dehiscence-related genes *INDEHISCENT (IND)/HECATE 3 (HEC3), REPLUMLESS (RPL)* and *SPATULA (SPT)/ALCATRAZ (ALC)* in *E. pusilla*, and found that the key players of the eudicot fruit regulatory network appear well-conserved in monocots. Protein-protein interaction studies revealed that MADS-domain complexes comprised of FRUITFULL (FUL), SEPALLATA (SEP) and AGAMOUS (AG) /SHATTERPROOF (SHP) orthologs can also be formed in *E. pusilla*, and that the expression of *HEC3, RPL*, and *SPT* can be associated with dehiscence zone development similar to Arabidopsis. Our expression analysis also indicates differences, however, which may underlie fruit divergence.

## Introduction

**Figure 1:**
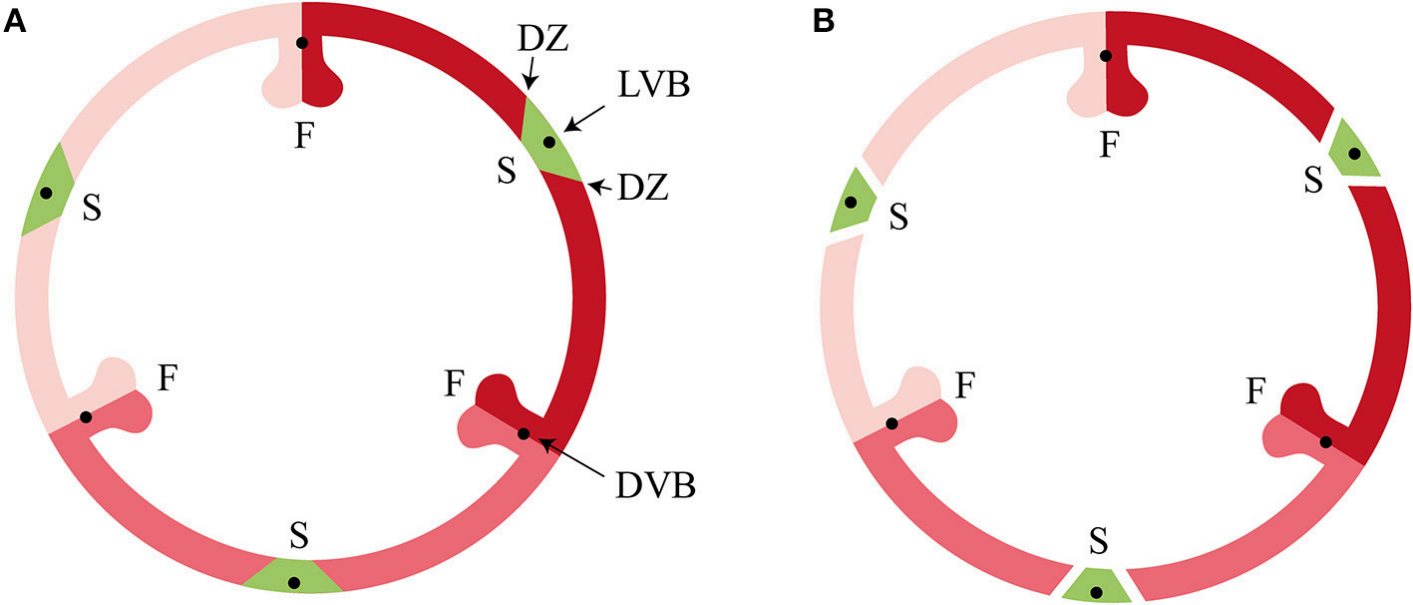
Tricarpellate orchidaceous fruit according to Brown (1831) and Rasmussen and Johansen (2006). **(A)** Immature fruit, **(B)** Mature fruit. DVB, dorsal vascular bundle; DZ, dehiscence zone; F, fertile valve; LVB, lateral vascular bundle; S, sterile valve. Color codes: red, fertile valve; green, sterile valve. (Illustrations by Erik-Jan Bosch).

Developmental mechanisms driving fruit diversification are still poorly understood, despite progress in the study of fruit formation in model plant species such as Arabidopsis (*Arabidopsis thaliana* (L.) Heyhn) and tomato (*Solanum lycopersicum* L.) (Gu et al., 1998; Ferrandiz et al., 1999; Vrebalov et al., 2009; Pabon-Mora and Litt, 2011). Research in monocots so far has focused mainly on cereal species such as rice, maize and wheat, all of which have relatively simple indehiscent fruits that consist of a one-layered pericarp. This leaves a big gap in the knowledge about evolution and development of fruits of other monocots, especially of the orchid family. Orchids are known for their spectacular floral diversity, but they also exhibit a large variety of fruit morphologies and dehiscence types (Brown, 1831; Beer, 1863; Dressler, 1993; Rasmussen and Johansen, 2006). Orchid fruits are very diverse in size and shape, but almost all share the same basic pattern and variation results from specific differentiation and development of the carpels. Orchid flowers are epigynous with an inferior ovary composed of three fused carpels containing many tiny ovules. After pollination the inferior ovary further develops into a dehiscent or indehiscent fruit. The three fused carpels develop into six valves: three fertile valves with a placenta, bearing the ovules, and three sterile valves. The origin and nature of these valves has been debated since the beginning of the nineteenth century. Rasmussen and Johansen (2006) presented the “split-carpel model” of the orchidaceous ovary, giving an explanation of the hexamerous pattern. According to this model, a typical orchid ovary consists of three sterile valves (located at the sepal bases) and three fertile valves (located at the petal bases), each consisting of two carpel-halves (Figure 1). According to Horowitz (1901), the main difference in morphology of the fertile and sterile valves is the size and/or number of cells. Cells of sterile valves do not become much larger during fruit maturation, whereas cells of fertile valves expand considerably. This would be in agreement with the split carpel model, where the fertile valves are the actual carpels, while the sterile valves are structures containing the mid-nerves descending from the sepals. Almost all orchids have dehiscent dry fruits, with a few exceptions, such as the berries of *Neuwiedia zollingeri* Rchb. f. (Kocyan and Endress, 2001) and fleshy pods of *Vanilla pompona* Schiede (Pridgeon et al., 1999). Fruit development of dry dehiscent orchid fruits has been studied in *Oncidium flexuosum* Sims (Mayer et al., 2011), which led to the identification of a special layer of cells involved in fruit dehiscence. However, a detailed description of dehiscent fruits from other orchid species is lacking, as well as any molecular data about the genes that underlie fruit development in orchids. MADS-box genes have been shown to play an important role in fruit development, maturation, and ripening in several angiosperm species, among which the dry fruit species Arabidopsis and the fleshy fruit species tomato. However, whether there is a conserved regulatory network operating at the base of dry and fleshy fruit development is still unclear. In dry dehiscent fruits of Arabidopsis, the MADS-domain proteins AGAMOUS (AG), SHATTERPROOF 1/2 (SHP1/2) and FRUITFULL (FUL) are essential for carpel formation (AG), as well as for fruit development and dehiscence (SHP and FUL) (Gu et al., 1998; Ferrandiz et al., 2000; Ferrandiz and Fourquin, 2014). FUL represses *SHP1/2* expression in the valves of the fruit, which ensures proper dehiscence zone development (Ferrandiz et al., 2000). SHP1/2 in their turn are expressed in the valve margins, where they activate the expression of *INDEHISCENT (IND)* and *ALCATRAZ (ALC)*, which are required for separation of the valves and the formation of a lignified cell layer initiating this separation (Rajani and Sundaresan, 2001; Liljegren et al., 2004). *REPLUMLESS (RPL)* is expressed in the replum at the other side of the valve margin and controls the development of the Arabidopsis replum by repression of SHP1/2 (Roeder et al., 2003). Both RPL and FUL are necessary for the proper development of a functional dehiscence zone in Arabidopsis fruits by repressing *SHP* expression in the valve margins (Ballester and Ferrandiz, 2017). In addition, the MADS-domain factors SEPALLATA 1-3 (SEP1-3), which promote higher-order complex formation, are also highly active in the Arabidopsis fruit and can interact with AG, SHP, and FUL (De Folter et al., 2004, 2005). Orthologs of these MADS-domain factors have been shown to play important roles in fruit development and ripening in tomato (Vrebalov et al., 2009; Bemer et al., 2012; Ferrandiz and Fourquin, 2014). Thus, complexes consisting of homologs of the MADS-domain proteins AG, SHP, FUL, and/or SEP seem to be generally important for fruit development in the eudicots. Whether the same MADS-domain factors play a role in orchid fruit development is still unclear, but a recent study from Lin et al. (2016) revealed that there are several MADS-box genes expressed in mature *Erycina pusilla* (L.) N.H. Williams & M.W. Chase fruits, pointing to a role for MADS-box genes in orchid fruit development as well. There is no data available yet about the presence and activity of homologs of the downstream target genes *IND/HECATE3* (*HEC3*), *SPATULA (SPT)/ALC* and *RPL* in orchids. Homologs of these Arabidopsis genes have also been found to be expressed in fruits of different Solanaceae species, suggesting that their role may be more broadly conserved. To increase our knowledge of fruit anatomy in different orchid species and of the molecular gene regulatory network that underlies fruit development in orchids, we undertook a combined approach, in which we performed a detailed anatomical and molecular analysis of fruit development of the orchid species *E. pusilla*, which has dry dehiscent fruits*. Erycina pusilla* belongs to the subfamily Epidendroideae and subtribe Oncidiinae, and is a fast growing, small sized epiphytic orchid species occurring in the wild in South America with a relatively short life cycle. It develops from seed to flowering stage in less than a year and is an upcoming model system for orchid research (Pan et al., 2012; Chou et al., 2013; Lin et al., 2013, 2016; Lee et al., 2014; Dirks-Mulder et al., 2017). To expand the study of fruit divergence in the orchid family, we compared development and dehiscence of *E. pusilla* fruits with those of fruits from the terrestrial species *Epipactis helleborine* (L.) Crantz (subfamily Epidendroideae, tribe Neottieae from Europe, Asia and North-Africa) and *Cynorkis fastigiata* Thouars (subfamily Orchidoideae, tribe Orchideae from Madagascar and surrounding islands). To investigate whether the regulatory network underlying fruit development in eudicots could to some extent be conserved in orchids, we investigated the fruit-expressed MADS-box genes in *E. pusilla* by performing detailed expression analysis and determining the protein-protein interactions of the proteins encoded by these genes. In addition, we performed expression analysis of close homologs of other well-known Arabidopsis fruit genes to investigate to what extent the genetic network driving fruit patterning and lignification of Arabidopsis corresponds to that of *E. pusilla*.

## MaterialS and Methods

### Plant Material

A more than 20 year old inbred line of *E. pusilla* originally collected in Surinam was grown in climate rooms under controlled conditions (7.00–19.00 h light regime), at a temperature of 22°C and a relative humidity of 50%. The orchids were cultured *in vitro* under sterile conditions on Phytamax™ orchid medium with charcoal and banana powder (Sigma-Aldrich) with 4 g/L Gelrite™ (Duchefa) culture medium. Pollination was conducted manually by placing the pollinia of flowers on each other's stigma. The seeds were ripe after 14–16 weeks and subsequently sown into containers (Duchefa) using sterile fresh culture medium. Fruits were collected from this laboratory strain of *E. pusilla* at 0, 1, 3, 5 days after pollination (DAP) and 1, 2, 3, 4, 8, and 12 weeks after pollination (WAP). Ripe seeds were collected from open fruits, after 16 WAP. Fruits of *E. helleborine* and *C. fastigiata* were collected at different developmental stages in the Hortus botanicus (Leiden, The Netherlands). Different developmental stages were determined by assessing the relative degradation of the floral remains and the size of the fruits.

### Fixation for Micromorphology

Fruits were fixed with standard formalin aceto-alcohol [FAA: 50% ethanol; 5% glacial acetic acid; 5% formalin (Sigma-Aldrich and Boom)] for 1 h under vacuum conditions at room temperature. They were placed on a rotating platform for 16 h (at room temperature). The fruits were washed once in 70% ethanol and subsequently stored in 70% ethanol at room temperature.

### London Resin (LR)—Polyhydroxy-Aromatic Acrylic Resin—White Embedding

Fruits, stored in 70% ethanol, were cut off transversally and rinsed in absolute ethanol for 1 h. Subsequently, the fruits were incubated in the following solutions: 8 h in 3:1 absolute ethanol in LR White (SPI supplies, Pennsylvania); overnight in 2:1 absolute ethanol in LR White overnight; 8 h in 1:1 absolute ethanol in LR White; overnight in 1:2 absolute ethanol in LR White; 8 h in 1:3 absolute ethanol in LR White and lastly overnight in LR White. Gelatin capsules (Electron Microscopy Sciences) were filled with the tissue and fresh LR White and placed in an oven at 60°C for 48 h.

### LR White Sectioning

LR White resin embedded samples were sectioned using a Leica RM2265 microtome (Leica Biosystems, Germany). The samples were trimmed until the tissue of interest was reached. Using a tungsten knife (Leica), at a 4°angle, sections of 5 μm thickness were obtained. The sections were placed in a drop of 40% acetone on a microscope slide. The slides were placed on a hot plate at 70°C for at least 1 h, after which they were stained.

### Staining, Visualization, Valve Area, and Cell Layer Measurements

LR white embedded sections were stained for 2 min with a solution of 0.2% Toluidine Blue and 0.2% Borax in distilled water, rinsed with distilled water, placed on a hot plate at 50–60°C for 20 s and mounted with Entellan mounting medium (Merck-Millipore). The slides were scanned using Bright field and Z-stacking on a 2D Scanning Panoramic Viewer 250 (LUMC, Leiden, The Netherlands). Scanned slides were viewed and analyzed with Case Viewer software (3DHISTECH). Areas of individual fertile- and sterile valves of the fruits were measured using Case Viewer software (3DHISTECH). For the reliability of the valve area measurements, the valve areas of cross-sections of the fruits were used as the metric for fruit size. These areas are quite robust against distortions and angle under which the anatomical slides were made, and highly reproducible. For *E. pusilla* the areas of the fertile and sterile valves were determined using the perimeter of these valves in fruits of 0 DAP, 5 DAP, 7 DAP, 2 WAP, 5 WAP, 8 WAP, 11 WAP, 15 WAP, and 16 WAP. Per time point, at least three fruits obtained of the same inbred laboratory strain were used. Of these fruits, six sections, three fertile and sterile valves per section, were measured. The number of cell layers of the fruit walls of all three orchid species was determined from at least 4 different fruits. Counts were performed in quarto for 3–4 slides per fruit. Cell number was determined in the same sections for 6–9 valves per developmental stage. Cells in the vascular bundles and placental tissues were not included.Handmade cross sections of *E. pusilla, E. helleborine*, and *C. fastigiata* fruits, stored in 70% ethanol, were stained with 1% phloroglucinol (Sigma-Aldrich) in 96% ethanol for 1 h. The cross sections were subsequently washed with 25%-(v/v) hydrochloric acid (HCl) (Sigma-Aldrich) and immediately examined under a Binocular microscope (Zeiss SteREO Discovery.V12).

### X-Ray Micro-Computed Tomography (Micro-CT)

Fruits were infiltrated with 1% phosphotungstic acid (Brunschwig) in 70% ethanol for 3–4 days, where PTA solution was refreshed daily. Scans were performed on a Zeiss Xradia 510 Versa 3D. Data were stacked and processed with Dragonfly Pro 2.0 (Object Research Systems, Montreal Canada).

### Scanning Electron Microscopy (SEM)

Fruits were dehydrated twice for 20 min in 90% ethanol and twice for 20 min in absolute ethanol. The fruits were dried using liquid carbon dioxide (CO_2_) with a Leica EM CPD300 critical point dryer (Leica Microsystems, Wetzlar Germany). Dried fruits were then placed on a stub with Leit-C conductive carbon cement (Neubauer) and spray-coated with 20 nm of Platinum/Palladium in a Quorum Q150TS sputter-coater. Fruits were observed with a JEOL JSM-7600F field emission scanning electron microscope.

### Transmission Electron Microscopy (TEM)

Dehiscence zones of *E. pusilla* fruits were cut and fixed with Karnovsky fixative (2% formaldehyde and 2.5% gluteraldehyde) for 3 h on a rotating platform at 4°C and 2 h post-fixed with 1% osmium tetroxide (OsO_4_) in the dark, both in 0.1 M sodium cacodylate buffer (pH 7.2). Samples were stained and dehydrated with a 1% uranyl acetate replacement (UAR) (Electron Microscopy Sciences) in 30% ethanol and dehydrated in an ascending 1% UAR ethanol series of 50-70-96 for 10 min each, twice with absolute ethanol for 20 min and once with acetonitrile for 20 min each, all on a rotating platform. They were embedded in epoxy-resin (48% EMbed-812, 21% dodecenyl succinic anhydride, 29% methyl-5-norbornene-2,3-dicarboxylic anhydride [Electron Microscopy Sciences) and 2% benzyl dimethylamine (Agar Scientific)] through a graded series of epoxy-resin:acetonitrile; 1:2, 1:1 both for 1 h, 1:1 overnight, 2:1 for 1 h and 100% epoxy-resin for 3 h. The submerged samples were placed in a vacuum for 20 min. Epoxy-resin was placed in molds and placed under vacuum for 20 min after which the samples were polymerized in an oven for 48 h at 60°C. Ultra-thin sections of 70 nm were cut with Leica Ultracut-S (Leica Co. Ltd) and directly mounted on copper grids (G2010-Cu, Electron Microscopy Sciences). The grids were rinsed in triple distilled water for 20 min and stained with 4% UAR for 20 min in the dark. The grids were subsequently rinsed 3 times with distilled water for 30 s. They were stained a second time with lead citrate (Electron Microscopy Sciences) according to Reynolds (1963). Images were made using the JEM-1400plus transmission electron microscope (JEOL Ltd).

### RNA Extraction, cDNA Synthesis, and Quantitative Real-Time PCR

Total-RNA was extracted from two different pools of fruits and seeds of the same inbred laboratory strain of *E. pusilla* using the RNeasy Plant Mini Kit. Two biological replicates were used in this study of *E. pusilla*, as the variation in expression of developmental genes between individuals is negligible. Extracted RNA was treated with DNase I, Amp Grade (Invitrogen 1U/μl) to digest single- and double-stranded DNA following the manufacturer's protocol. cDNA was synthesized with up to 1 μg of DNase-treated RNA using iScript™ cDNA Synthesis Kit (Bio-Rad Laboratories) following the manufacturer's protocol. A positive control (CTRL) and a no reverse transcriptase (NRT) control were included. Beacon Designer™ (Premier Biosoft, www.oligoarchitect.com) software was used to design primers (Table S3). Quantitative real-time PCR was performed using the CFX384 Touch Real-Time PCR system (Bio-Rad Laboratories) and iQ™ SYBR® Green Supermix (Bio-Rad Laboratories). The reaction mixture contained 1x iQ™ SYBR® Green Supermix, 0.2 μM of each primer, 1 ng cDNA template (triplicate reactions) for each target gene and from a fruit time-point for two sets of isolated RNA (six reactions in total). For each amplicon group, a positive control was included (= CTRL, RNA extracted from *E. pusilla* flower buds), a negative control (= NTC, reaction mixture without cDNA) and a no reverse transcriptase treated sample (= NRT, control sample during the cDNA synthesis). For all the qPCR reactions, the amplification protocol was as follows: initial denaturation of 5 min 95°C followed by 20 s 95°C; 30 s 61°C; 30 s 72°C; plate read, for 50 cycles; followed by a melting curve analysis of 5 s, 65°-95°C with steps of 0.2°C to confirm single amplified products. Quantification Amplification results (QAR) were used for analysis with LinRegPCR (v2017.0, dr. J.M. Ruijter) (Ruijter et al., 2006, 2015).

### Yeast Two-Hybrid Analysis (Y2H)

A yeast two-hybrid screening was performed as described by De Folter et al. (2005); (De Folter and Immink, 2011). Full-length clones were used for the construction of the yeast two-hybrid vectors (Table S1).

### Protein Alignment and Phylogenetic Analysis

Nucleotide sequences of *SPT/ALC, IND/HEC3* and *POUNDFOOLISH (PNF)/RPL* genes were downloaded from NCBI GenBank (www.ncbi.nlm.nih.gov), OneKP (https://sites.google.com/a/ualberta.ca/onekp) and Phytozome (https://phytozome.jgi.doe.gov). Most of the orchid nucleotide sequences were downloaded from Orchidstra (orchidstra2.abrc.sinica.edu.tw) and belong to orthologous group ORGP07662 for the predicted *SPT* genes, ORGP11571 for the predicted *HEC3* genes (both Pfam ID00010, HLH) and ORGP08194 for the predicted *RPL* genes (Pfam ID07526, POX and PF05920, homeobox_KN). A multiple sequence alignment was performed using the ClustalW alignment tool within Geneious v7.1.5 (www.geneious.com), based on translated nucleotides), taking into account protein domains and amino acid motifs that have been reported as conserved for the three gene lineages by Pabon-Mora et al. (2014). Regions that did not align were removed prior to further analysis. For the visualization of the alignments, Bioedit (www.mbio.ncsu.edu/BioEdit/bioedit.html) was used. Phylogenetic trees were generated with the Geneious Tree Builder plug-in using the Maximum likelihood (ML) method with gymnosperm gene lineages as out-group based on Pabon-Mora et al. (2014). Numbers above the branches represent bootstrap support values from 100 replicates.

## Results

### Description of *Erycina pusilla* Fruit Development

**Figure 2:**
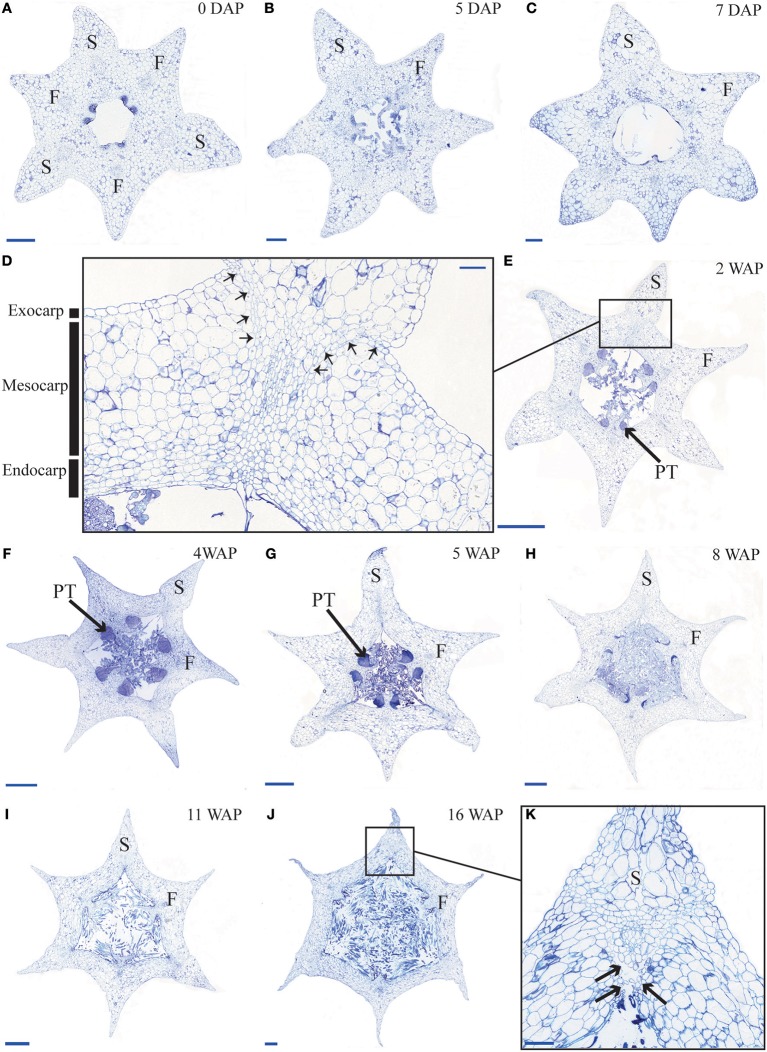
Time-line of developing *E. pusilla* fruit cross sections, embedded in LR White and stained with toluidine blue. **(A)** 0 DAP. **(B)** 5 DAP. **(C)** 7 DAP. **(D)** Magnified part of the sterile valve at 2 WAP. Arrows indicate the dehiscence zone. Black boxes the exo-, meso- and endocarp layer. **(E)** 2 WAP. **(F)** 4 WAP. **(G)** 5 WAP. **(H)** 8 WAP. **(I)** 11 WAP. **(J)** 16 WAP. **(K)** Magnified part of the sterile valve at 16 WAP. Black arrows indicate the dehiscence zone. DAP, days after pollination; WAP, weeks after pollination; F, fertile valve; S, sterile valve; PT, pollen tube. Scale bar **(A–C,K)** = 0.2 mm, **(D)** = 0.1 mm, **(E–I)** = 1 mm, **(J)** = 0.5 mm.

**Figure 3:**
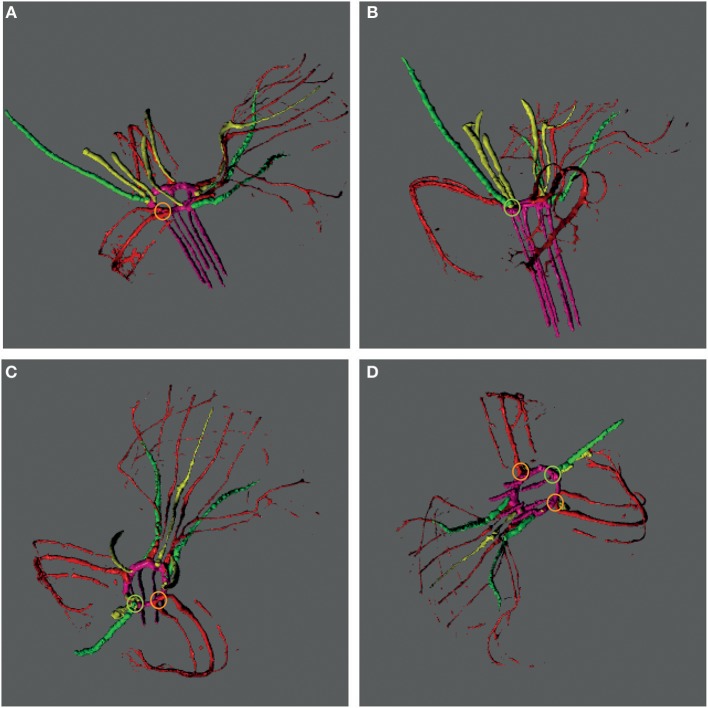
Vascular bundle patterns in an *E. pusilla* fruit and wilted flower at 5 DAP visualized with a micro-CT reconstruction and depicted at different angles. **(A)** Lateral view of the fruit and wilted flower from the right hand side. **(B)** Posterior view of the fruit and wilted flower. **(C)** Apical view of the fruit with the wilted labellum projected upwards. **(D)** Inferior view of the fruit with the wilted labellum projected downwards. Color codes: green, vascular bundles in sepals; red, vascular bundles in petals; pink, plexus and vascular bundles in fruit; yellow, vascular bundles in stamens, stelidia, and callus; Orange circle, connection of a dorsal vascular bundle with a petal; Green circle, connection of a lateral vascular bundle with a sepal.

To obtain more insight into the development of orchid fruits we documented changes in anatomy and morphology during fruit maturation of *Erycina pusilla*, which develops a dry dehiscent capsule, a very common fruit type for the orchid family (Dressler, 1993). Fruit development starts around one day after pollination (1 DAP) and the fruit reaches its final size at 16 weeks after pollination (16 WAP) (Figure S1). From 16 WAP onwards, the valves of the *E. pusilla* fruit slowly separate longitudinally, starting from the apex, but remaining fused at the base. The fruits usually open along three of the six fusion zones and split into three wide seed-bearing fertile valves whereas the narrow sterile valves remain connected to one of the fertile valves (Figure S1H). To examine the development of the different fruit tissues and the dehiscence zone in more detail, we investigated the *E. pusilla* fruits using light microscopy (LM), Scanning Electron Microscopy (SEM), Transmission Electron Microscopy (TEM), and X-ray micro computed tomography (micro-CT). Figure 2 shows sections of the different fruit stages observed with LM. At 0 DAP the ovary consists of three sterile and three fertile valves, which form broad protrusions. The fertile valves contain two zones with small cells that will develop into the placenta (Figure 2A). In the first week after pollination, the fruits slowly expand while the ovules start to develop from the placenta of the fertile valves (Figures 2B,C). The valves each consist of three major fruit tissue layers: exocarp, mesocarp and endocarp. The outer exocarp is single layered; the mesocarp, or middle part of the fruit wall, has multiple cell layers consisting of parenchyma cells and the innermost endocarp consists of a few cell layers from which the placenta differentiates in the fertile valves (Figure 2D). At 2 WAP, specific cell files can be clearly observed at both sides of the sterile valve, marking the initiation of three V-shaped dehiscence zones. These zones emerge in the first week after pollination at the boundaries of the sterile and fertile valves, and consist of a layer of small cells (Figures 2D,E). In orchids, the signal to initiate fruit maturation is provided by pollination rather than fertilization. Fertilization of the ovules inside the ovary takes place much later; this can vary from a few days up to months, depending on the circumstances and orchid species (Arditti, 1992; Chen and Fang, 2016; Chen et al., 2016; Fang et al., 2016). For *E. pusilla* six bundles of pollen tubes, at each side of the placenta, develop around 2 WAP and increase in diameter until 4 WAP, when fertilization probably takes place and seed development starts (Figures 2D,E and Figures S2A,B). The pollen tube bundles begin to shrink at 6 WAP, and have completely disappeared at 11 WAP (Figures 2F–I and Figures S2C,D), indicating that the ovules have been fertilized. At 16 WAP, the fruit opens where the endocarp borders the sterile valves. Opening progresses along the dehiscence zones at the boundaries of the fertile and sterile valves by rupture of the cells (Figures 2J,K). The main morphological changes observed during the development of the fruit of *E. pusilla* are summarized in Table 1. Micro-CT was used to visualize internal structures of entire *E. pusilla* fruits during development. Fruits older than 4 WAP were full of seeds, which made it difficult to visualize other internal structures. In these fruits, many umbilical cords (funiculi) could be detected. The funiculi were connected to the three main dorsal vascular bundles of the fertile valves and the developing ovules in the placenta regions (Movie S1). According to Österberg (1883) and Rasmussen and Johansen (2006), fertile valves are located at the bases of the petals and sterile valves at the sepal bases. By following the vascular bundles in a micro-CT scan of an *E. pusilla* fruit of 5 DAP from the base upward to the wilted floral organs at the apex, we found further support for this hypothesis (Figure 3). The six main vascular bundles in the fruit connect in a plexus (Figure 3, indicated in purple) situated between the base of the fruit and the apex with wilted floral organs. The dorsal vascular bundles are connected to a petal (orange circles, Figures 3A,C,D) and the lateral vascular bundles to a sepal (green circles, Figures 3B–D).

**Table 1:** The main morphological changes of *E. pusilla* fruits observed during development.

**Time (days/weeks)**	**Main morphological changes**
0 DAP−7 DAP	Elongation of the fruit
	Cell division in the sterile and fertile valves
	Trichome development
2 WAP−5 WAP	Increase of the volume of the fruit
	Cell division and growth in the fertile and sterile valves
	Development of six pollen tube bundles
	Formation of dehiscence zones
	Thickening of trichome walls
6 WAP−11 WAP	Increase of the volume of the cells in the sterile and fertile valves
	Shrinking of the pollen tube bundles
	Development of dehiscence zones
	Lignification of the trichomes
12 WAP−16 WAP	Shrinking of the fruit
	Disappearance of pollen tube bundles
	Lignification of the endocarp
	Dehiscence of the fruit

*DAP, days after pollination; WAP, weeks after pollination*.

**Figure 4:**
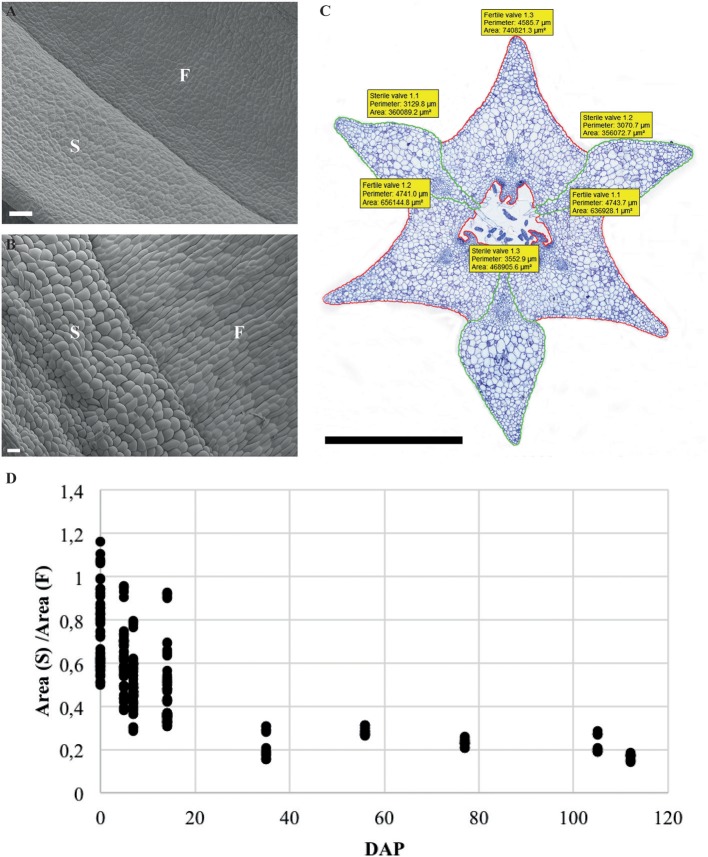
Ratio and normalized area of the fertile and sterile valve of *E. pusilla* fruits during development from 0 to 112 DAP. **(A)** SEM image of external surface of 1 WAP. **(B)** SEM image of external surface of 16 WAP. **(C)** Perimeter and area measurement of a fruit cross-section at 5 WAP. **(D)** Spread of the ratio area (S)/area (F). Each dot represents one individual fruit. F, fertile valve; S, sterile valve. DAP, days after pollination; Color codes: green, sterile valve; red, fertile valve. Scale bar **(A,B)** = 100 μm, **(C)** = 1 mm.

Cross-sections of fruits in different developmental stages of the terrestrial orchid species *C. fastigiata* and *E. helleborine* were compared with those of the epiphytic orchid species *E. pusilla* (Figure 2 and Figure S3). The number of cell layers is very constant throughout development for all three orchid species (Table S2). The fruit wall of *E. pusilla* consists of 13–19 cell layers at the narrowest parts of the fertile valve. The terrestrial species have fruits walls consisting of less cell layers, with 6–9 layers for *C. fastigiata* and 7–11 for *E. helleborine* (Table S2), both measured in the fertile valves, which is in agreement with the observations made by Beer (1863) that terrestrial species have thinner fruit walls. While the number of cell layers in the fruit wall is stable, anticlinal cell divisions are responsible for initially expansion of the *E. pusilla* fruit. Figures S4A,B show that the cells in the fertile valves divide frequently until 5 DAP, after which the cell division rate slows down. At 2 WAP, only occasional cell division events were still observed and the maximum number of cells in the fertile valves is reached around 4 WAP. Cell expansion in the fertile valve is initiated between 5 and 7 DAP (Figure S4C) and continues to contribute to fruit growth until 12–16 WAP (Figures 2F–J and Figure S4C), after which fruit size decreases again due to dehydration. Thus, the fertile valves of *E. pusilla* grow mainly by cell division between 0 and 5 DAP, by a combination of cell division and cell expansion between 5 DAP and 2 WAP, and by cell expansion after 2 WAP. In contrast to the fertile valves, only a limited number of cell divisions was observed in the sterile valves between 0 and 5 DAP (Figure S4A), after which cell division was terminated completely. Growth of the sterile valves between 0 and 5 DAP was mainly caused by cell expansion (Figure S4C), which was also responsible for further growth in the stages thereafter. However, the fertile valves appeared to expand to a much larger extent than the sterile valves. To investigate this in more detail, we determined growth of the different valves in fruits of *E. pusilla* by measuring the perimeter of the fertile and sterile valves during development and calculating the total area per valve (Figures 4A–D). Although the spread of the area measurements is high during the first two weeks of fruit development (Figure 4D), a clear increase of the fertile valve-area and a relative decrease of the sterile valve-area can be seen toward fruit maturation, confirming the hypothesis of Horowitz (1901) that increase in fruit volume of *E. pusilla* during development is mainly caused by expansion of the fertile valves.

### Dehiscence Zone Development in Fruits of Different Orchid Species

**Figure 5:**
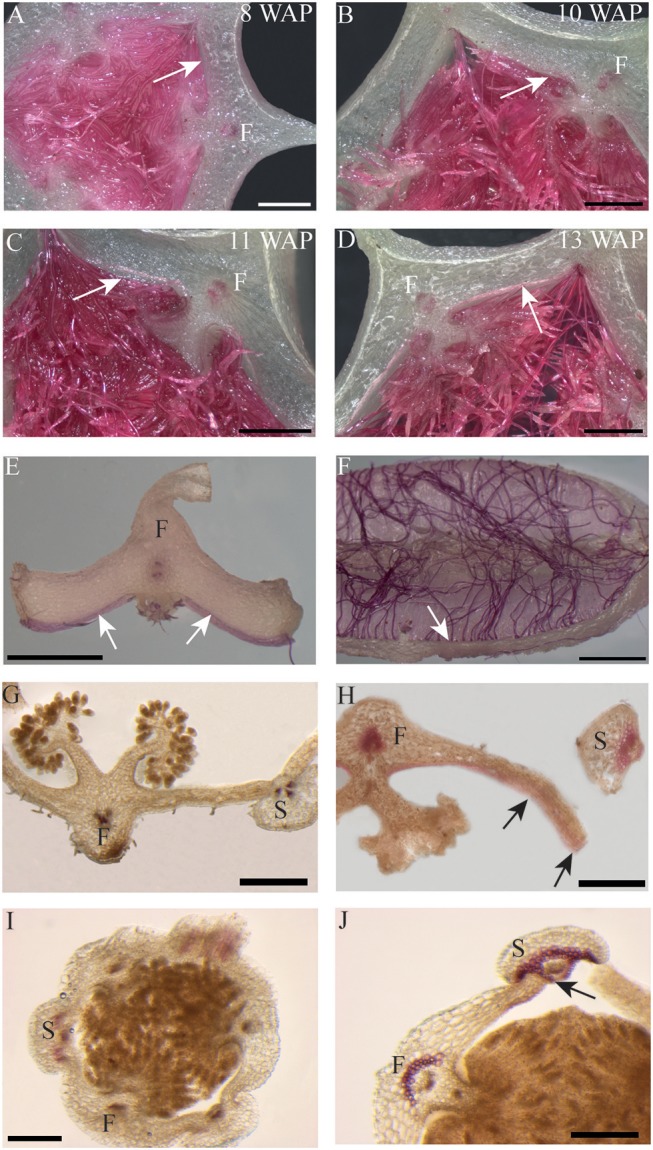
Phloroglucinol staining of fruit cross-sections. *Erycina pusilla*: **(A)** 8 WAP. **(B)** 10 WAP. **(C)** 11 WAP. **(D)** 13 WAP. **(E,F)** Dehisced fertile fruit valve. *Epipactis helleborine*: **(G)** Unripe and indehisced fruit. **(H)** Ripe and dehisced fruit. *Cynorkis fastigiata*: **(I)** Unripe and indehisced fruit. **(J)** Ripe and dehisced fruit. F, Fertile valve; S, sterile valve. White and black arrows indicate lignified endocarp. Scale bar **(A–D,G,H)** = 1 mm. **(E,F)** = 2 mm. **(I,J)** = 0.5 mm.

**Figure 6:**
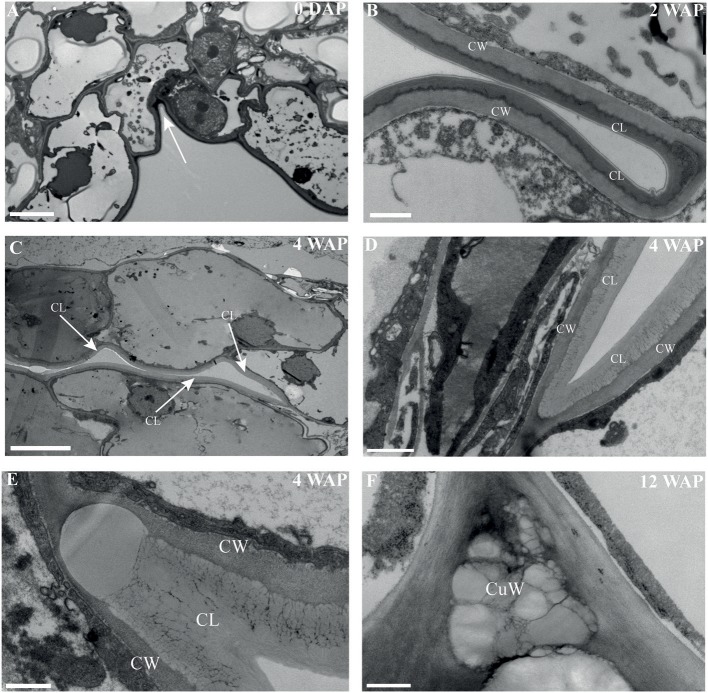
An electron-dense, cuticular lipid layer developing in the dehiscence zone of *E. pusilla* fruits visualized with TEM. **(A)** 0 DAP, white arrow pointing to the incision between a fertile and a sterile valve. Cuticle-like layer in the dehiscence zone **(B)** 2 WAP. **(C)** 4 WAP. White arrows point out the direction of the developing cuticle-like layer in the dehiscence zone. **(D,E)** Detailed image at 4 WAP. **(F)** 12 WAP. CW, cell wall; CL, cuticular-like layer; CuW, cuticular-like wax. Scale bar **(A)** = 5 μm, **(B,D)** = 1 μm, **(C)** = 10 μm, **(D,E)** = 500 nm.

**Figure 7:**
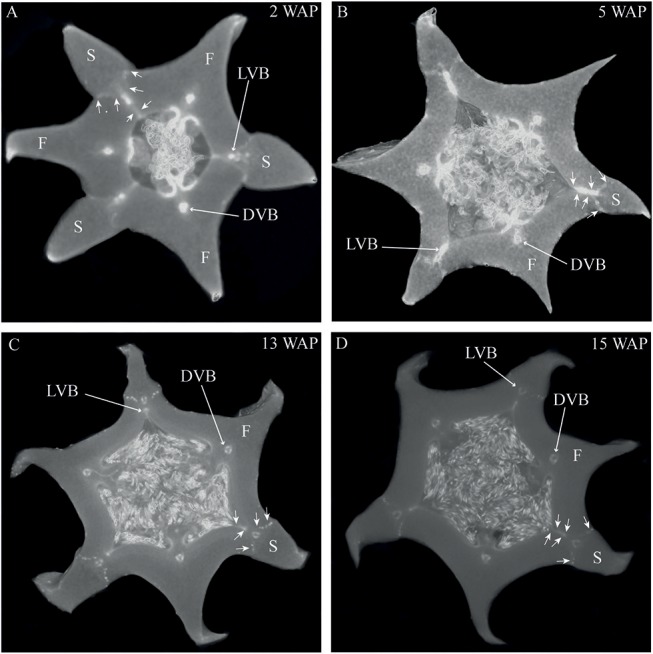
Cross sections of 3D reconstructions of micro-CT scans of *E. pusilla* fruits stained with PTA. **(A)** 2 WAP. **(B)** 5 WAP. **(C)** 13 WAP. **(D)** 15 WAP. Long arrows indicate one vascular bundle of a fertile or sterile valve. Short arrows indicate the location of the dehiscence zone between a fertile and a sterile valve. VB, vascular bundle; F, fertile valve; S, sterile valve. (No scale bars can be included for 3D images).

The most characteristic aspect of the development of dry dehiscent fruits is the formation of the dehiscence zone. In dry fruits of Arabidopsis, specific cell files in the valve margin become lignified toward maturation and the fruit splits at a cell file adjacent to this lignified layer, called the separation layer (Ferrandiz et al., 1999; Ferrandiz, 2002; Liljegren et al., 2004). The innermost endocarp layer also becomes lignified in Arabidopsis. In the Solanaceae, lignification occurs in dehiscent dry fruit species as well, but the extent and location is variable in different species (Pabon-Mora and Litt, 2011). To investigate lignification of *E. pusilla* fruits, we stained sections with phloroglucinol at different developmental stages. Lignin in the cell walls of the vascular bundles in the valves was stained with phloroglucinol. Until 6 WAP there was no sign of lignification anywhere besides the vascular bundles. Staining was observed in the trichomes, which appeared at 5 DAP in the endocarp on each side of the fertile valves, but became lignified around 8 WAP. The innermost endocarp cell layer also became lignified, which was evident around 10 WAP (Figures 5A–D). During the entire development of the fruit there was no sign of lignification of the dehiscence zones at the boundaries of the fertile and sterile valves of *E. pusilla*. This lignification pattern is similar to the one observed for the fruits of the epiphytic species *O. flexuosum* Sims (Mayer et al., 2011). To investigate dehiscence zone development in dry dehiscent fruits of other orchid species, we also performed phloroglucinol staining of fruits from the terrestrial species *E. helleborine* and *C. fastigiata*. Interestingly, different patterns were observed for these species. The fertile valves of *E. helleborine* became lignified during development along the entire endocarp layer (Figures 5G–H), whereas lignification was absent in the fertile valves of *C. fastigiata* (Figures 5I,J). Lignification of the dehiscence zones of *E. helleborine* was not evident, but staining of three ripe fruits did reveal some lignification at the sites of separation between the sterile and fertile valves (Figure 5H). By contrast, the entire V-shaped dehiscence zone, including the small innermost endocarp cell layer of the sterile valves, became lignified in *C. fastigiata* toward maturation of the fruit (Figure 5J). While no lignification of the dehiscence zones was observed in the fruits of *E. pusilla*, ultra-structural observations revealed the formation of an electron-dense, possible cuticular lipid-layer (Figure 6). This layer developed from the exocarp starting from an invagination between the fertile and the sterile valve (Figure 6A) and expanded during fruit development toward the endocarp (Figures 6B,C). At higher magnification polysaccharide fibers were visible (Figures 6D,E), known to be often present in cuticle layers (Figure 6F) (Fernandez et al., 2016). In micro-CT sections taken from 3D-models of *E. pusilla* fruits in different developmental stages, the vascular bundles of each of the six valves were clearly visible (Figure 7). Interestingly, additional stained cells could be observed between each fertile and sterile valve. These files have a similar location as the developing layer of small cells observed with LM (Figure 2D) and the lipid-layer observed with TEM (Figures 6B,C). The differential contrast of these cells indicates that their chemical content differs from that of their neighboring cells, and thus strengthens the idea that cells at the sterile-fertile valve border produce a yet uncharacterized substance instead of lignin prior to dehiscence.

### Gene Expression Changes During Orchid Fruit Development

**Figure 8:**
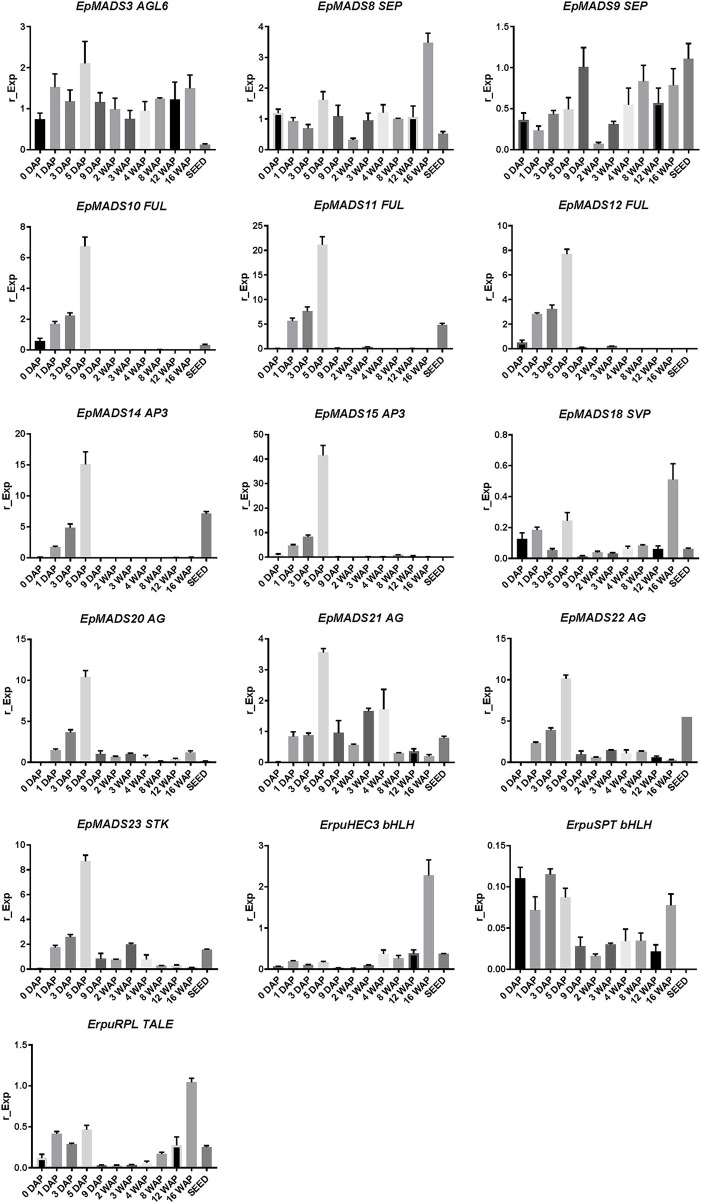
Fruit specific expression patterns of selected MADS-box gene copies in *E. pusilla* of *AGL6, SEP, AP1*/*FUL, AP3, SVP, AG, STK*, and three fruit specific genes *SPT, HEC3, and RPL*. Each graph shows the relative expression during twelve stages of development. Expression of the genes was normalized to the geometric mean of three reference genes *Actin, UBI2*, and *Fbox*. Each column shows the relative expression of two cDNA pools of different fruits of the same inbred laboratory strain, both tested in triplicate. DAP, days after pollination; WAP, weeks after pollination. Y-axis: relative gene expression. The error bars represent the Standard Error of Mean.

**Figure 9:**
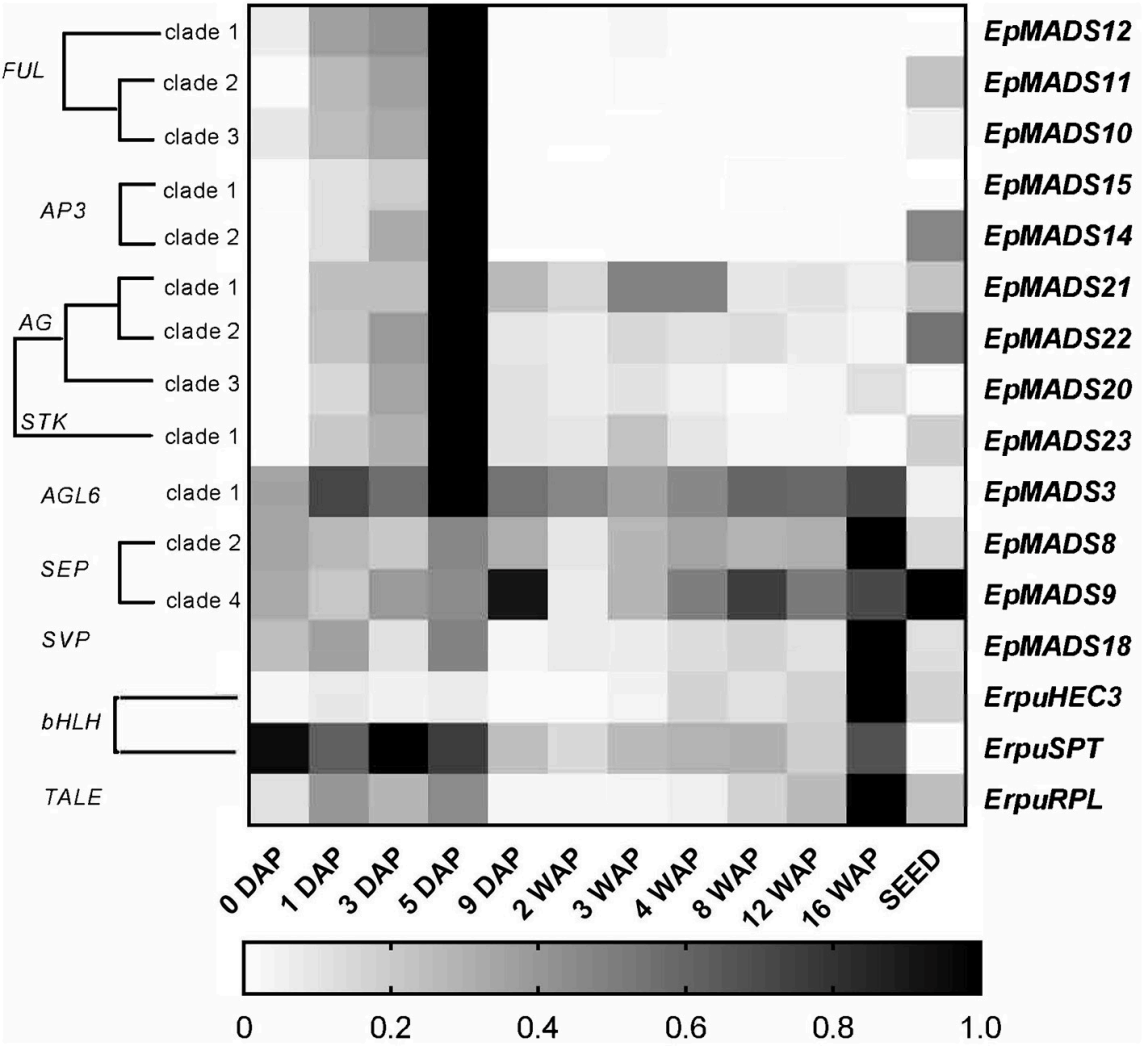
Heat map representation of expression of developmental genes in *E. pusilla* fruits and ripe seeds. The *FUL-, AP3-, AG-, STK-, AGL6-, SEP-, SVP-, bHLH-*, and *TALE-like* copies were retrieved from different gene lineage clades during eleven stages of fruit development. Expression of the genes was normalized to the geometric mean of the reference genes *Actin, Fbox*, and *UBI2*. The scale for each gene was set to 1 for the highest value. DAP, days after pollination; WAP, weeks after pollination.

To obtain a better understanding of the molecular mechanisms driving development and dehiscence of *E. pusilla* fruits, a detailed expression study was performed on MADS-box genes known to be expressed in fruits of this orchid species (Lin et al., 2016) together with homologs of two bHLH-like genes (*HEC3* and *SPT*) and the homeodomain transcription factor (*RPL*), which are part of the Arabidopsis fruit gene regulatory network (Ferrandiz et al., 2000; Ferrandiz, 2002; Girin et al., 2011). Eleven different developmental stages of *E. pusilla* fruits were used, as well as a separate sample of seeds from mature fruits, to determine in which phases of fruit development the different genes may be functioning. To confirm whether orchid *RPL, HEC3* and *SPT* sequences, downloaded from different databases were the correct orthologs, they were aligned according to Pabon-Mora et al. (2014). With these alignments, ML analyses were performed. The orchid protein sequences downloaded all aligned well with other SPT (Figure S6 and Table S5), HEC3 (Figure S7 and Table S6), and RPL (Figure S8 and Table S7) homologs. Our ML analysis yielded trees with considerable up to high (62–99%) BS support for the orchid clades of SPT (Figure S9), HEC3 (Figure S10) and RPL (Figure S11). The *AGL6-like* gene copy *EpMADS3* and the *SEP-like* genes *EpMADS8* and -*9* were expressed throughout fruit development (Figures 8, 9), in line with their putative role in the formation of various higher order complexes. At 16 WAP, *EpMADS8* was clearly more highly expressed than in the other fruit stages (Table S4). The other MADS-box genes showed more specific expression patterns. The *AP1/FUL*-*like* genes *EpMADS10*, −*11 and* −*12* all showed a major increase of expression between 0 and 5 DAP, while their expression could hardly be detected after this stage. This expression pattern was also observed for the *AP3-like* genes *EpMADS14* and *EpMADS15*, the *AG-like* genes *EpMADS20* and *EpMADS22*, and the *STK-like* gene *EpMADS23*. The *AG* homolog *EpMADS21* displayed a different expression pattern, with substantial expression until 4 WAP, while also having a peak at 5 DAP. Both *EpMADS21* and *EpMADS22* were still expressed around 16 WAP, when dehiscence was initiated. Interestingly, the *SHORT VEGETATIVE PHASE* homolog *EpMADS18* was also expressed in the fruit, with the highest expression at 16 WAP, indicating that it may contribute to the initiation of dehiscence. Overall, the *E. pusilla fruit*-expressed MADS-box genes showed predominantly high expression during fruit patterning, but *EpMADS18* and *EpMADS8* were also clearly expressed at 16 WAP, when the *AG* homologs *EpMADS21* and *EPMADS22* also still exhibited expression (Figures 8, 9 and Table S4). The expression patterns of the *SVP* homolog *EpMADS18* and *EpRPL* were quite similar: expression between 0 and 5 DAP, a decrease in expression between 1 and 12 WAP and a significant increase in fruits at 16 WAP. The *bHLH-like* gene *SPT* showed a similar expression pattern, although it was also moderately expressed between 5 DAP and 12 WAP. This indicates that these genes may both be involved in fruit patterning as well as in the initiation of dehiscence. The bHLH-like gene *HEC3* was mainly expressed after 4 WAP with a significant increase at 16 WAP. These expression data suggest that all three homologs of Arabidopsis fruit specification genes are also important for the development of orchid fruits (Figures 8, 9 and Table S4).

### MADS-Box Protein-Protein Interaction During Orchid Fruit Development

To investigate whether the fruit regulatory network present in Arabidopsis (Dinneny et al., 2005) and tomato (Bemer et al., 2012) could be conserved in orchids, we performed yeast two-hybrid experiments with fruit-expressed MADS-domain proteins from *E. pusilla*. Full-length proteins were fused to the GAL4 binding domain or activation domain and all baits and preys were screened against each other. The auto-activation test revealed that EpMADS8, EpMADS21, and EpMADS22 exhibited auto-activation, and these proteins were therefore only tested as prey (fused to the activation domain). The results of the yeast two-hybrid analysis are summarized in Table 2. Figure S5 shows the growth of the spotted yeast colonies. The AP1/FUL protein EpMADS11 showed a similar interaction pattern compared to the Arabidopsis FUL-protein and could also form homo-dimers like AtFUL. EpMADS10 did not interact at all. EpMADS18, an SVP-like protein, formed homo-dimers and bound to most of the proteins used in this study. No interaction was found between members of the AG- and STK clades. Two AP3 proteins, EpMADS14 and−15 did not interact at all with the MADS-box proteins used in this study.

**Table 2:** Yeast two-hybrid screening of interactions between MADS-box proteins of *E. pusilla* and Arabidopsis FUL (AtFUL).

**Arabidopsis homolog**	***E. pusilla***	**EpMADS3**	**EpMADS8**	**EpMADS9**	**EpMADS10**	**EpMADS11**	**EpMADS12**	**EpMADS14**	**EpMADS15**	**EpMADS18**	**EpMADS20**	**EpMADS21**	**EpMADS22**	**EpMADS23**	**AtFUL**
AGL6	EpMADS3														
SEP	EpMADS8		X												
SEP	EpMADS9														
AP1	EpMADS10														
FUL	EpMADS11														
FUL	EpMADS12														
AP3	EpMADS14														
AP3	EpMADS15														
SVP	EpMADS18														
AG	EpMADS20														
AG	EpMADS21											X			
AG	EpMADS22												X		
STK	EpMADS23														
AtFUL	AtFUL														

*Light gray, Interaction in one direction only (either with AD or BD). Dark gray, Interaction in both directions. White, No interactions. X, not analyzed*.

## Discussion

### Conservation and Divergence of Fruit Anatomy Within the Orchid Family

According to Beer (1863), epiphytic and terrestrial orchid fruits are thought to develop differently regarding fruit wall thickness. We found that both fruits of the terrestrial orchid species *C. fastigiata* and *E. helleborine* do indeed have relatively thinner walls than fruits of the epiphytic orchid species *E. pusilla* (Figure 2 and Figure S3). Fruits of terrestrial and epiphytic orchids differ in the number of cell layers in the exocarp, resulting in thin-walled terrestrial fruits and thick-walled epiphytic fruits, possibly to protect the latter against the much more fluctuating moisture and UV radiation levels present in tree canopies as compared with the forest floor. Several models of fruit dehiscence have been described for angiosperms with parietal fruits: (i) loculicidal dehiscence: each locule splits at the middle of each carpel while the septa remain intact (ii) septicidal dehiscence: each septum, bordering a locule, splits in two and (iii) septifragal dehiscence: each placental region with its adjacent valves breaks away from the sterile (septal) region. The orchid species we investigated all showed septifragal dehiscence, whereby each carpel consisted of two halves of fertile valves and one sterile valve (Figure 1) as described by Brown (1831). During the entire development of the fruit there was no sign of lignification of the dehiscence zone at the boundaries of the fertile and sterile valves of *E. pusilla*. *Oncidium flexuosum*, another epiphytic member of the Oncidiinae, showed the same lignification pattern as *E. pusilla* and the valves of this orchid also separated longitudinally along a dehiscence zone consisting of small cells (Mayer et al., 2011). Using osmium tetroxide as a secondary fixative, not only enhanced the contrast but it also increased the retention of lipids in the tissue (Hayat, 1970). With a two-step staining procedure with UAR followed by lead citrate (Reynolds, 1963) in TEM, we could visualize the development of a cuticular lipid layer in *E. pusilla* fruits. Cross sections of 3D reconstructions of micro-CT scans of *E. pusilla* fruits, stained with phosphotungstic acid (PTA), a negative staining agent for lipids (Melchior et al., 1980; Bello et al., 2010), also revealed a white-stained layer at the dehiscence zones of the valves of *E. pusilla* (Figure 7). On the other hand, in both terrestrial species a layer of lignified cells can be seen at the valve margins (Figures 5H,J). The major components of plant cuticles are cutin and cuticular wax (Yeats and Rose, 2013). Both are primarily composed of fatty acid derivates. Renault et al. (2017) described a lignin-related biochemical pathway in mosses, which is responsible for the formation of cuticles. These authors found that cutin and lignin have the same evolutionary origin whereby the enzyme CYP98 (from the family of cytochromes P450) plays an important role in either the production of lignin in seed plants and in the development of a phenol-enriched cuticle in mosses. If indeed there is a common ancestor of lignin, cutin and suberin (wax) polymers, this suggests that orchids use different strategies for the dehiscence of their fruits. Cutin synthase plays an important role in the cutin biosynthesis pathway and belongs to the GDSL-lipase family, which is widely represented in orchid transcriptomes. We found more than 75 possible hits for *E. pusilla* in the Orchidstra database (orchidstra2.abrc.sinica.edu.tw). Which of these proteins are responsible for the formation of the cuticular lipid layer at the dehiscence zone of *E. pusilla* fruits is still unknown and deserves further investigation.

### Fruit Molecular Networks Appear to be Partly Conserved Between Eudicots and Orchids

**Figure 10:**
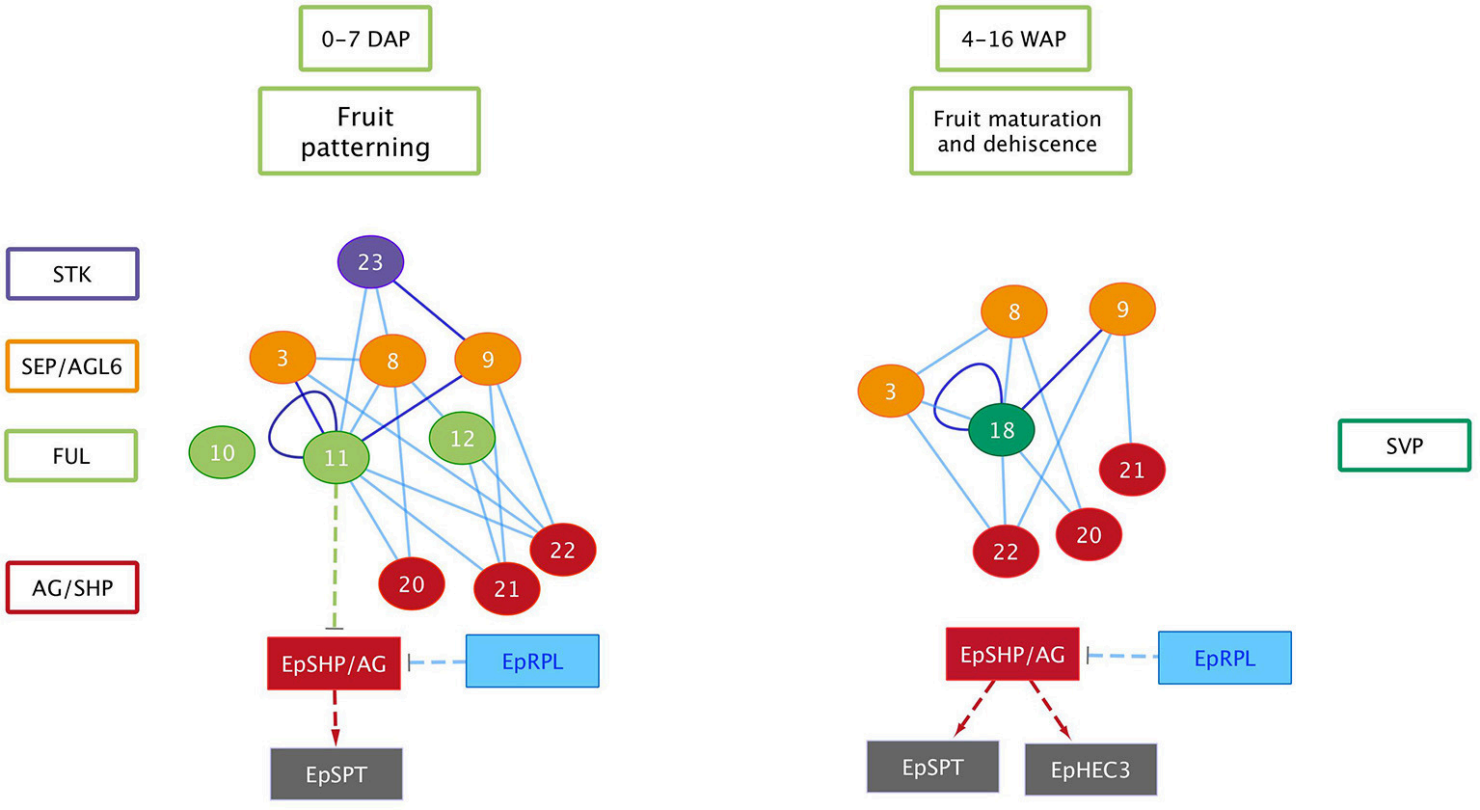
Orchid fruit developmental protein and gene network for *E. pusilla*. Circles, MADS-box proteins; rectangles, genes; solid lines, validated protein–protein interactions (blue: one direction, purple: both directions); dashed arrows: putative activation interactions; dashed T-bars: putative repression.

We show in this study that orchid homologs of well-known Arabidopsis fruit development genes are also expressed during orchid fruit development, and that the encoded MADS-domain transcription factors are able to form dimeric complexes with a similar composition as the Arabidopsis complexes. However, there were also distinct differences. For example, two *AP3*-clade members were found to be expressed during orchid fruit development. This might be correlated with the fact that floral remains stay attached to the developing fruit in orchids, whereas in tomato, pepper and thale cress these remains fall off after fruit maturation has been initiated. The *FUL* homologs *EpMADS11* and *EpMADS12* are expressed during fruit patterning, suggesting that they may regulate the initiation and specification of the fruit in the cell division stage. Remarkably, both genes are not expressed during later stages when the dehiscence zone is specified. This suggests that this mechanism is regulated without the contribution of *FUL-like* genes, which would be different from the situation in Arabidopsis. In line with protein-protein interaction data from Arabidopsis (De Folter et al., 2005) and tomato (Leseberg et al., 2008), the FUL-homologs EpMADS11 and EpMADS12 are both able to interact with the AG homologs EpMADS21 and EpMADS22. EpMADS11 also interacts with the third C-class gene EpMADS20. Because the split of the C-class genes *AG* and *SHP* occurred at the base of the core Eudicots (Kramer et al., 2004; Zahn et al., 2006), it is not possible to distinguish the three *E. pusilla* C-class genes based on their sequence identity. However, distinct expression of *EpMADS21* and *EpMADS22* in the seeds suggests that these genes could function as SHP homologs. In addition to interacting with C-class proteins, the FUL ortholog EpMADS11 can also interact with all three SEP/AGL6 homologs, while EpMADS12 interacts with the SEP homolog EpMADS8 only. Interestingly, *EpMADS10*, which belongs to the *AP1/FUL* clade and is highly expressed in fruits at 5 DAP, does not interact with any other fruit-expressed MADS proteins. A closer inspection of the EpMADS10 protein sequence unveiled a deletion of a 9 amino acids domain at the border of the I- and K-domains. Because both regions are essential for the dimerization of MADS-domain proteins (Van Dijk et al., 2010), this deletion is a probable cause for the lack of interactions found (Table 2 and Figure S5). We also could not detect any interactions for the AP3 homologs EpMADS14 and−15, which form obligate heterodimers with PISTILLATA (PI) homologs (Bartlett et al., 2016) in most species. The *AP3* homolog *EpMADS13* and *PI* homolog *EpMADS16* are hardly expressed in orchid fruits (Lin et al., 2016). It is good to note however, that both PI and AP3 did not show any interactions in Arabidopsis yeast two-hybrid studies (De Folter et al., 2005) despite ubiquitous proof that they are interacting *in planta*, suggesting that false negative data may easily be obtained for these proteins. Based on the combination of our expression and interaction data, it is very likely that an AG/SHP-SEP-FUL regulatory module is also acting during *E. pusilla* fruit development. The FUL paralogs EpMADS11 and EpMADS12 can both interact with SEP and AG/SHP homologs, but EpMADS12 exhibits a more specific interaction pattern and may have sub-functionalized after duplication. To compare the interaction capacity of the *E. pusilla* FUL homologs with that of Arabidopsis FUL (AtFUL), we also tested AtFUL against the fruit-expressed *E. pusilla* MADS-domain proteins, and found that the interaction profile of AtFUL was highly similar to that of *EpMADS11*, indicating conservation of protein structure and function. Orchids disperse their seeds by different ways using either dehiscent or indehiscent fruits and by various vectors such as wind, water or animals. Here we propose a fruit developmental network for the dry dehiscent fruit of the orchid species *E. pusilla* based on the expression data and the protein-protein interactions data obtained in this study (Figure 10). Our model is based on the fruit core genetic regulatory network from Arabidopsis (Ferrandiz et al., 2000; Ferrandiz, 2002; Roeder et al., 2003). Based on our results, there is a clear difference between the regulators involved in fruit patterning and fruit maturation and dehiscence in *E. pusilla*. During fruit patterning, FUL-like proteins interact with AG/SHP-like proteins and SEP-like proteins, thereby possibly regulating downstream targets that are also involved in Arabidopsis fruit development, such as SPT. *RPL* is also moderately expressed in this stage and may suppress the transcription of *AG/SHP* similar to Arabidopsis. During maturation and dehiscence, the MADS-box genes are less active, except for the E-class homologs *EpMADS8* and *EpMADS9* and the SVP homolog *EpMADS18*. In addition, homologs of the Arabidopsis dehiscence-zone specifiers *SPT, HEC3*, and *RPL* are all highly expressed, indicating that they perform a more prominent role in the last stage of fruit development, when the dehiscence zone is formed. *HEC3* and *RPL* are only expressed during late fruit development, which is different from the Arabidopsis fruit regulatory network. RPL may suppress the expression of *HEC3* and *SPT* in the orchid fruit regulatory network. In conclusion, the expression and protein-protein interaction data that we present here suggests that orchid fruit development may be regulated via a similar regulatory network as Arabidopsis fruit development. However, more functional data are required to validate our orchid fruit regulatory model, such as knockout studies of fruit-expressed transcription factors using for instance CRISPR/Cas9 mutagenesis, in combination with *in situ* hybridization studies for higher spatial resolution. These need to be performed in *E. pusilla* and other orchid species with different dehiscence patterns, to determine a general regulatory network for orchid fruits as well as the main molecular factors that are responsible for divergence in dehiscence zone formation.

## Author Contributions

AD-M, ES, MB, and BG designed the research and wrote the paper. AD-M, IA, LK, NM, JS, AvW, AdW, MvW, JZ, RB, BvH, JK, RL, WS, and MB performed the research. AD-M, IA, MuhB, LK, JS, AvW, MvW, JZ, RH, and MB analyzed the data.

### Conflict of Interest Statement

The authors declare that the research was conducted in the absence of any commercial or financial relationships that could be construed as a potential conflict of interest.

## Supplementary Material

Figure S1 Development of *E. pusilla* fruits from unfertilized ovary to mature fruit with ripe seeds. **(A)** Ovary. **(B)** Fruit 1 DAP. **(C)** Fruit 5 DAP. **(D)** Fruit 1 WAP. **(E)** Fruit 2 WAP. **(F)** Fruit 3 WAP. **(G)** Fruit 4 WAP. **(H)** Dehisced fruit 16 WAP. **(I)** Ripe seeds of dehisced fruit. DAP, days after pollination; WAP, weeks after pollination; Fl, flower; Cr, carpel; Fr, fruit, F; fertile valve; and S, sterile valve. Scale bars **(A–G)** = 5 mm, **(H)** = 10 mm and **(I)** = 1 mm.

Figure S2 Time-line of developing *E. pusilla* fruit cross sections of one of the fertile valves. **(A)** 2 WAP. **(B)** 5 WAP. **(C)** 8 WAP. **(D)** 11 WAP. Dashed circles indicate the left pollen tube bundle located in the fertile valve. Scale bar **(A)** = 0.2 mm, **(B–D)** = 0.5 mm.

Figure S3 Time-line of developing *Cynorkis fastigiata* and *Epipactis helleborine* fruits, embedded in LR White and stained with toluidine blue. **(A–F)** *C. fastigiata*. **(G–I)***. E. helleborine*. Scale bars **(A,C–F,I)** = 0.2 mm, **(B)** = 0.1 mm, **(G,H,J)** = 0.5 mm, **(K,L)** = 1 mm. F, fertile valve; S, sterile valve.

Figure S4 Growth of *E. pusilla* fruits by cell division and cell elongation. **(A)** Graph showing the number of cells in different growth stages. Cell number was determined in cross sections of fruits at different growth stages from at least 6 sterile and 6 fertile valves per growth stage. A trend line was drawn through the different data points. The error bars depict the SE. **(B)** Details of fertile valve sections showing the cell divisions with red asterisks. **(C)** The size of sterile and fertile valves in different growth stages. The detailed pictures (bottom) show the increase in cell size. Scale bar **(A–C)** = 0.2 mm.

Figure S5 Results of the yeast-two hybrid assay. Thirteen *E. pusilla* MADS-box proteins and one Arabidopsis protein (AtFUL) were screened against each other. After mating, the diploid yeast were grown for 5 days on SD medium lacking Leu, Trp, and His, supplemented with 5 mM 3-amino-1,2,4 triazole. Growth indicates an interaction between bait and prey.

Figure S6 Alignment of the bHLH domain of SPATULA/ALCATRAZ proteins based on Pabon-Mora et al. (2014) extended with orchid gene lineages. The bHLH was drawn based on Toledo-Ortiz et al. (2003) and corresponds with positions K359-Q410. Within the bHLH domain, black arrows indicate positions E13, R16, L27, K39, L56, which are conserved in all bHLH plant and animal genes. The H9 and R17 positions (red arrows) show amino acids that provide the SPT/ALC proteins with G-box (CACGTG) binding activity. Black dashed boxes: N-flank and C-flank showing the conserved motif LQLQVQ.

Figure S7 Alignment of the bHLH domain of HECATE3/INDEHISCENT proteins based on Pabon-Mora et al. (2014) extended with orchid gene lineages. The bHLH was drawn based on Toledo-Ortiz et al. (2003) and corresponds with positions N462-L515. Left black dashed box: N-flank of the bHLH domain: HEC domain (Kay et al., 2013) including domain 17 (Pires and Dolan, 2010). Right black dashed box: poorly conserved C-flank.

Figure S8 Alignment of the BELL-domain and the Homeo-domain of REPLUMLESS/POUNDFOOLISH (PNF) proteins based on Pabon-Mora et al. (2014) extended with orchid gene lineages. The BELL domain (Smith et al., 2002) and the conserved Homeodomain, based on Mukherjee et al. (2009) were drawn.

Figure S9 ML tree of the *SPATULA/ALCATRAZ* genes in seed plants. Branch colors denote the following taxa: Persian green, Gymnosperms; Blue, Basal angiosperms; Middle washed yellow, Monocots; Green, Basal eudicots; Purple, Core eudicots; Red, Brassicaceae. Bootstrap values are placed above the nodes.

Figure S10 ML tree of the *HECATE3/INDEHISCENT* genes in seed plants. Branch colors denote the following taxa: Persian green, Gymnosperms; Blue, Basal angiosperms; Middle washed yellow, Monocots; Green, Basal eudicots; Purple, Core eudicots; Red, Brassicaceae. Bootstrap values are placed above the nodes.

Figure S11 ML tree of the *REPLUMLESS/POUNDFOOLISH* genes in seed plants. Branch colors denote the following taxa: Persian green, Gymnosperms; Blue, Basal angiosperms; Middle washed yellow, Monocots; Green, Basal eudicots; Purple, Core eudicots; Red, Brassicaceae. Bootstrap values are placed above the nodes.

Movie S1 3D X-ray macroscopic reconstruction of a 3 WAP *E. pusilla* fruit. Umbilical cords (funiculi) can be detected between the vascular bundles of the fertile valves and the placenta regions. (No scale bar can be included for a 3D movie). WAP, week after pollination.

Table S1 AttB-primers used for the creation of inserts for Gateway cloning.

Table S2 Number of cell layers of *Erycina pusilla, Epipactis helleborine*, and *Cynorkis fastigiata* fruits during development. DAP, days after pollination; WAP, weeks after pollination; Cf, *Cynorkis fastigiata*; Eh, *Epipactis helleborine*.

Table S3 Transcript primer sequences and amplicon characteristics used for quantitative real-time PCR validation of the expression profiles of different transcripts, following MIQE guidelines (Bustin et al., 2009). The sequences listed here were downloaded from NCBI GenBank (www.ncbi.nlm.nih.gov), Orchidstra (orchidstra2.abrc.sinica.edu.tw) and our own fruit transcriptome dataset (Dirks et al., unpublished raw data).

Table S4 Difference in MADS-box gene expression between developmental stages of the fruit of *E. pusilla* as calculated using a variance analysis of measures using a Tukey multi-comparisons test. *P*-value style: GP: >0.05 (ns), < 0.05 (^*^), < 0.01 (^**^), < 0.001 (^***^), < 0.0001 (^****^). No value, No expression; DAP, days after pollination, WAP, weeks after pollination.

Table S5 Accession numbers of *SPT/ALC bHLH* transcription factor sequences used in the alignment. The Orchidaceae subfamilies are provided in parentheses.

Table S6 Accession numbers of *IND/HEC3* sequences used in the alignments and phylogenetic analyses. The Orchidaceae subfamilies are provided in parentheses.

Table S7 Accession numbers of *RPL/PNF* sequences used in the alignments and phylogenetic analyses. The Orchidaceae subfamilies are provided in parentheses.

## Abbreviations

*ALC*: *ALCATRAZ*
*AP1*: *APETALA1*
*AP3*: *APETALA3*
*AG*: *AGAMOUS*
*AGL6*: *AGAMOUS-like-6*
DAP: days after pollination
DZ: dehiscence zone
F: fertile valve
*FUL*: *FRUITFULL*
*IND*: *INDEHISCENT*
LM: light microscopy
*PNF*: *POUNDFOOLISH*
*RPL*: *REPLUMLESS*
*PI*: *PISTILLATA*
SEM: scanning electron microscopy
*SEP*: *SEPALLATA*
*SHP*: *SHATTERPROOF*
*SPT*: *SPATULA*
S: sterile valve
*SVP*: *SHORT VEGETATIVE PHASE*
TEM: transmission electron microscopy
VB: vascular bundle
Y2H: Yeast-two-Hybrid
WAP: weeks after pollination.
