# Supplementary figures and images for: Morphological and Molecular Characterization of Orchid Fruit Development
Source: Front Plant Sci. 2019 Feb 19;10:137. doi: 10.3389/fpls.2019.00137 (PMC6390509; doi:10.3389/fpls.2019.00137)

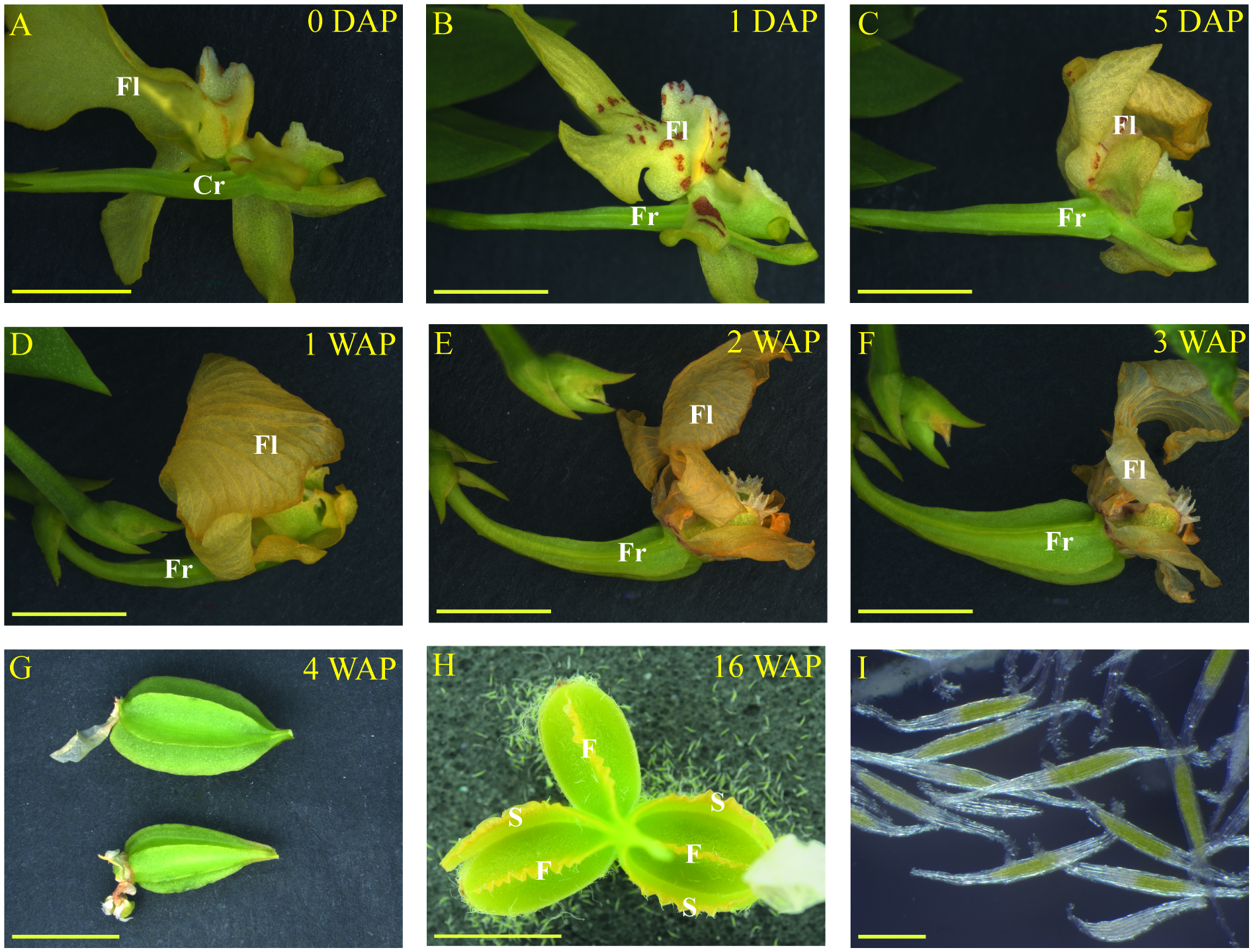

Supplement: Figure S1 — Development of E. pusilla fruits from unfertilized ovary to mature fruit with ripe seeds. (A) Ovary. (B) Fruit 1 DAP. (C) Fruit 5 DAP. (D) Fruit 1 WAP. (E) Fruit 2 WAP. (F) Fruit 3 WAP. (G) Fruit 4 WAP. (H) Dehisced fruit 16 WAP. (I) Ripe seeds of dehisced fruit. DAP, days after pollination; WAP, weeks after pollination; Fl, flower; Cr, carpel; Fr, fruit, F; fertile valve; and S, sterile valve. Scale bars (A–G) = 5 mm, (H) = 10 mm and (I) = 1 mm. [file Image_1.TIF]

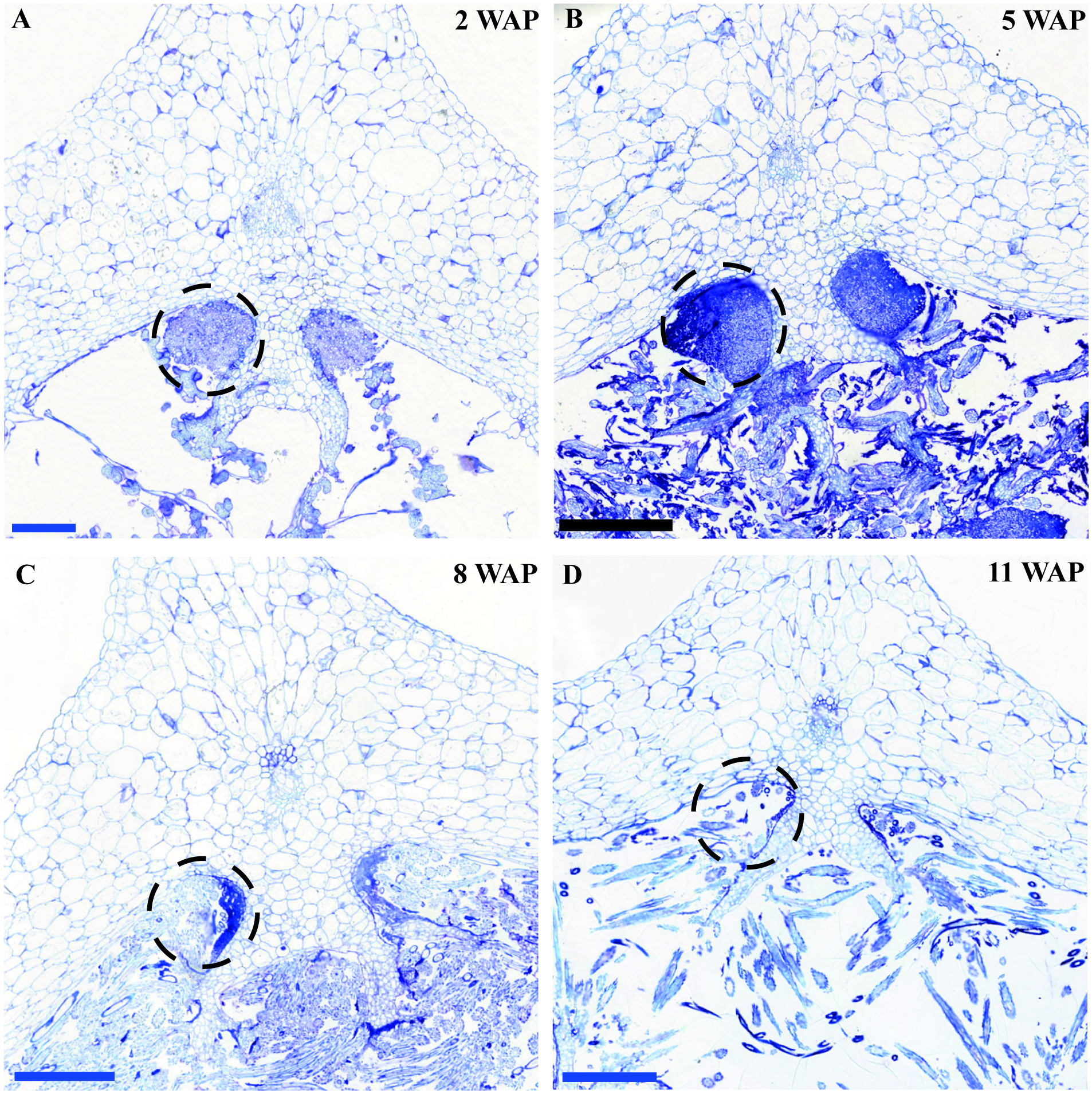

Supplement: Figure S2 — Time-line of developing E. pusilla fruit cross sections of one of the fertile valves. (A) 2 WAP. (B) 5 WAP. (C) 8 WAP. (D) 11 WAP. Dashed circles indicate the left pollen tube bundle located in the fertile valve. Scale bar (A) = 0.2 mm, (B–D) = 0.5 mm. [file Image_2.TIF]

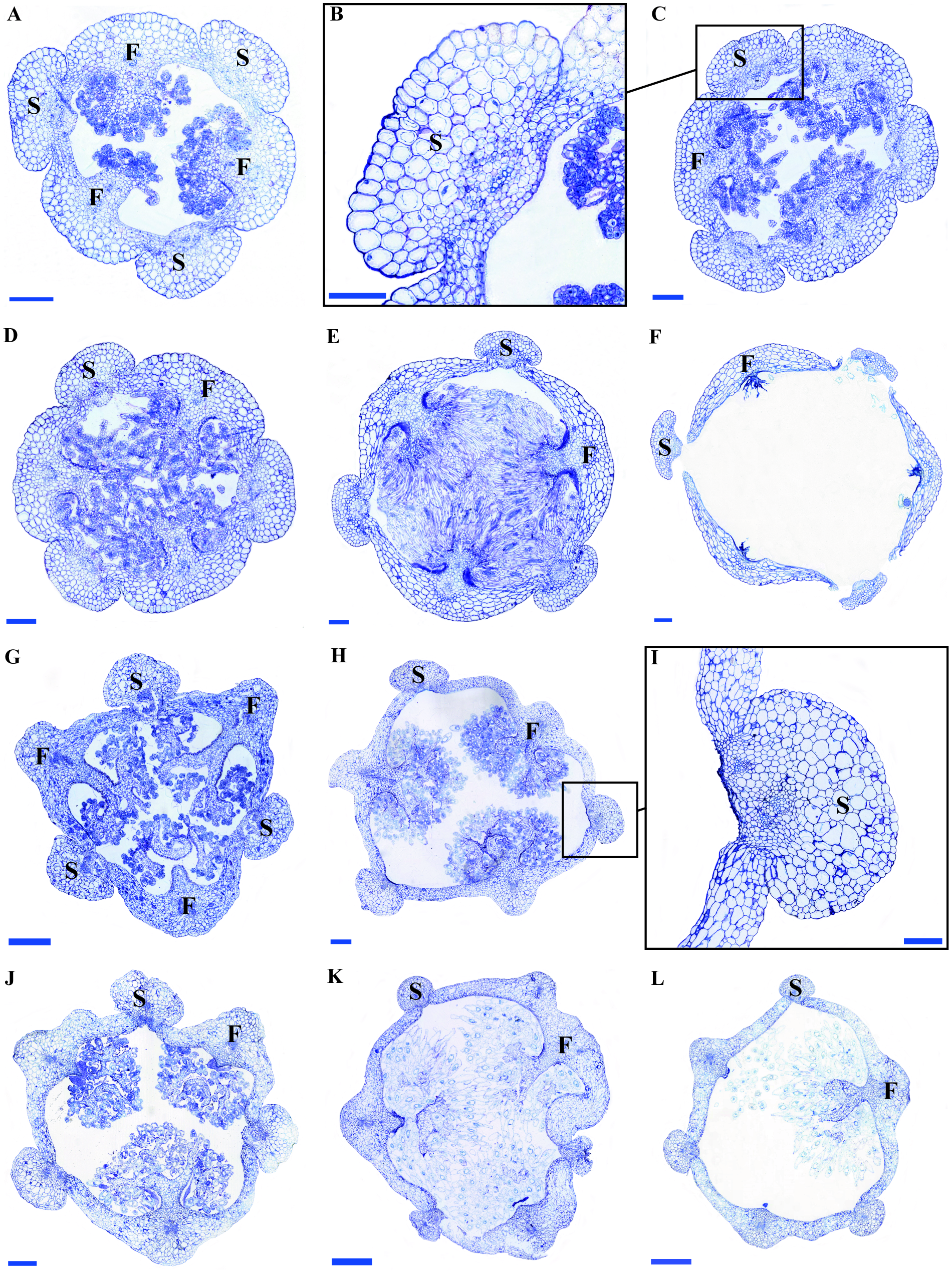

Supplement: Figure S3 — Time-line of developing Cynorkis fastigiata and Epipactis helleborine fruits, embedded in LR White and stained with toluidine blue. (A–F) C. fastigiata. (G–I). E. helleborine. Scale bars (A,C–F,I) = 0.2 mm, (B) = 0.1 mm, (G,H,J) = 0.5 mm, (K,L) = 1 mm. F, fertile valve; S, sterile valve. [file Image_3.TIF]

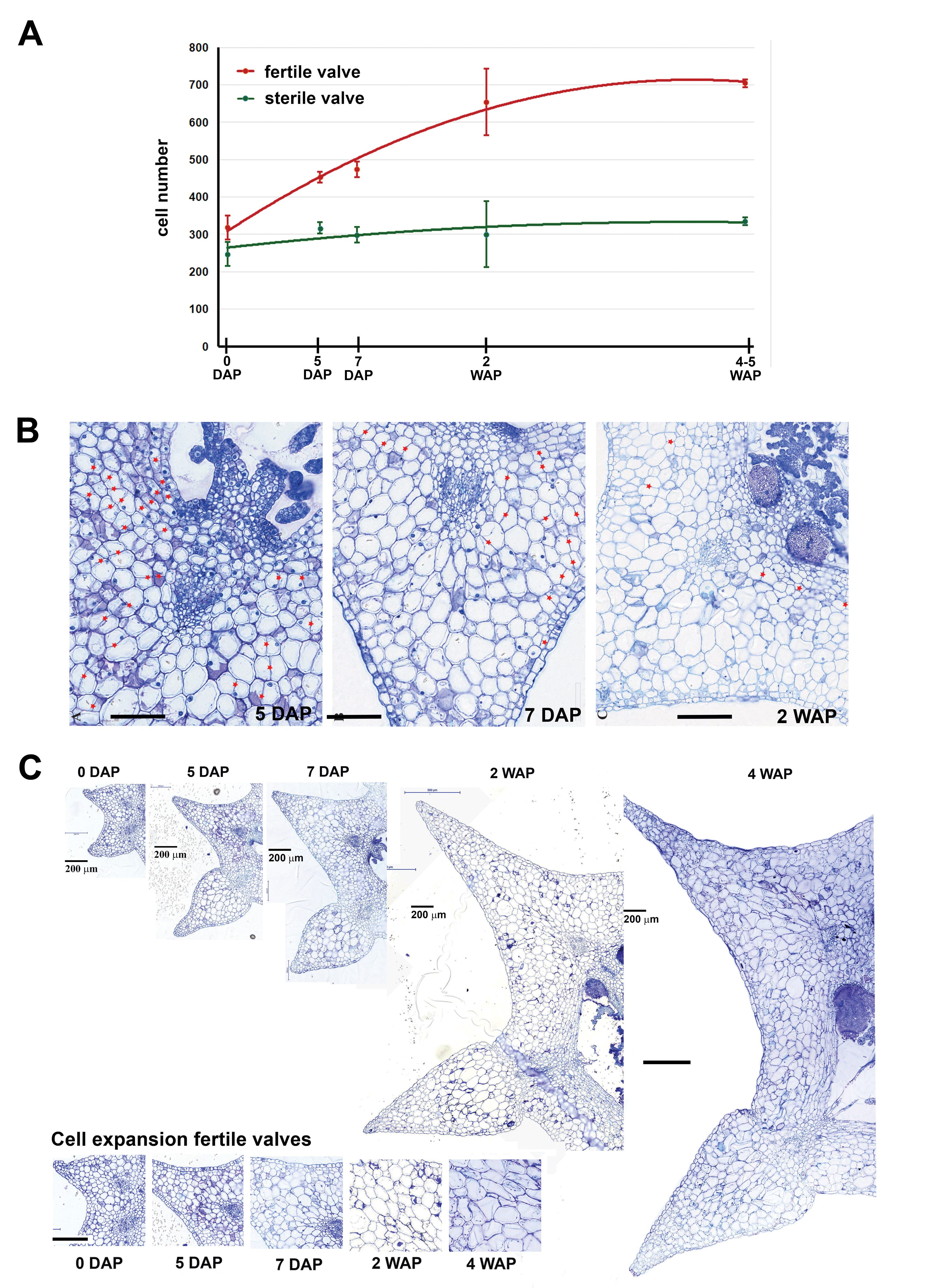

Supplement: Figure S4 — Growth of E. pusilla fruits by cell division and cell elongation. (A) Graph showing the number of cells in different growth stages. Cell number was determined in cross sections of fruits at different growth stages from at least 6 sterile and 6 fertile valves per growth stage. A trend line was drawn through the different data points. The error bars depict the SE. (B) Details of fertile valve sections showing the cell divisions with red asterisks. (C) The size of sterile and fertile valves in different growth stages. The detailed pictures (bottom) show the increase in cell size. Scale bar (A–C) = 0.2 mm. [file Image_4.JPEG]

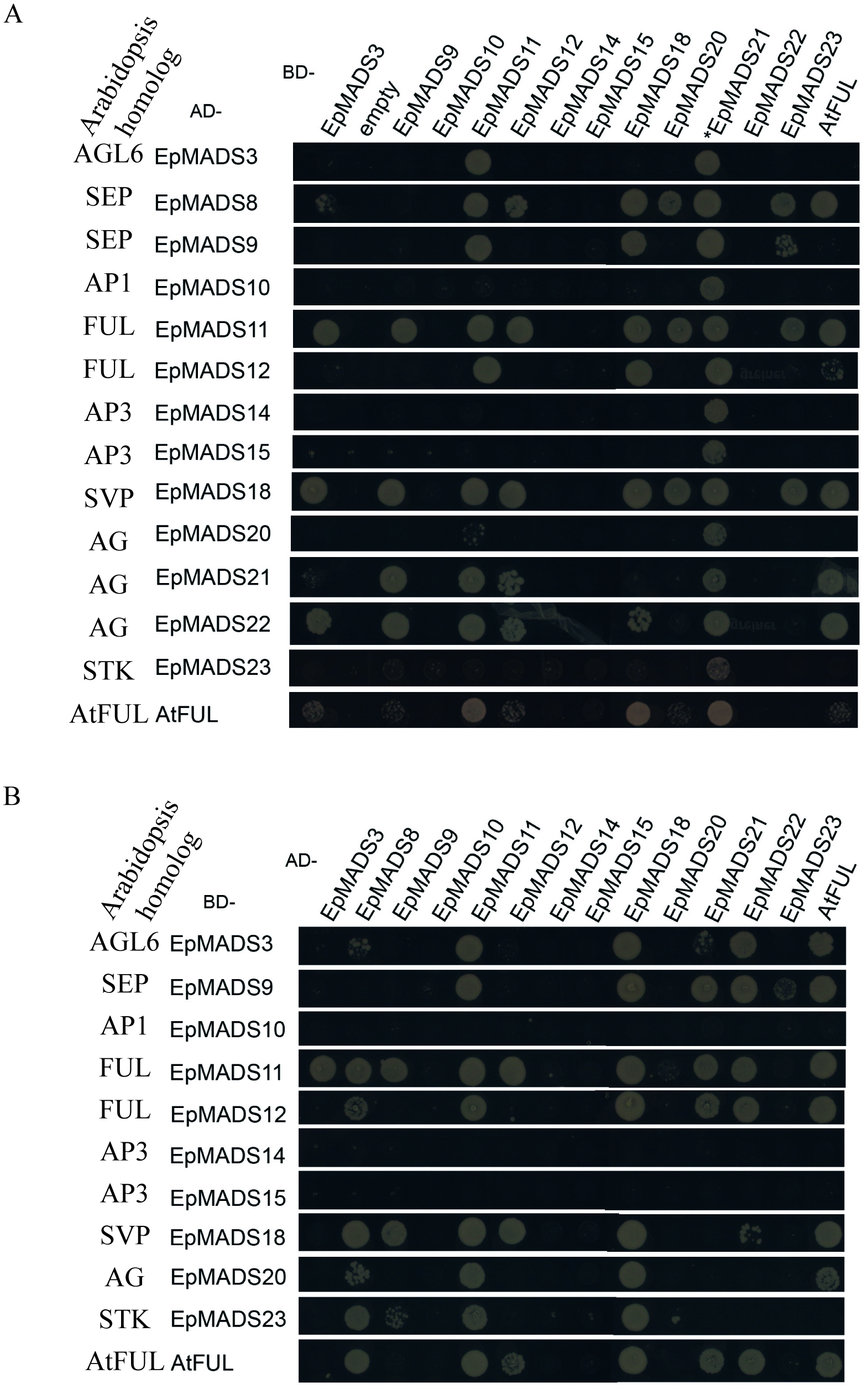

Supplement: Figure S5 — Results of the yeast-two hybrid assay. Thirteen E. pusilla MADS-box proteins and one Arabidopsis protein (AtFUL) were screened against each other. After mating, the diploid yeast were grown for 5 days on SD medium lacking Leu, Trp, and His, supplemented with 5 mM 3-amino-1,2,4 triazole. Growth indicates an interaction between bait and prey. [file Image_5.TIF]

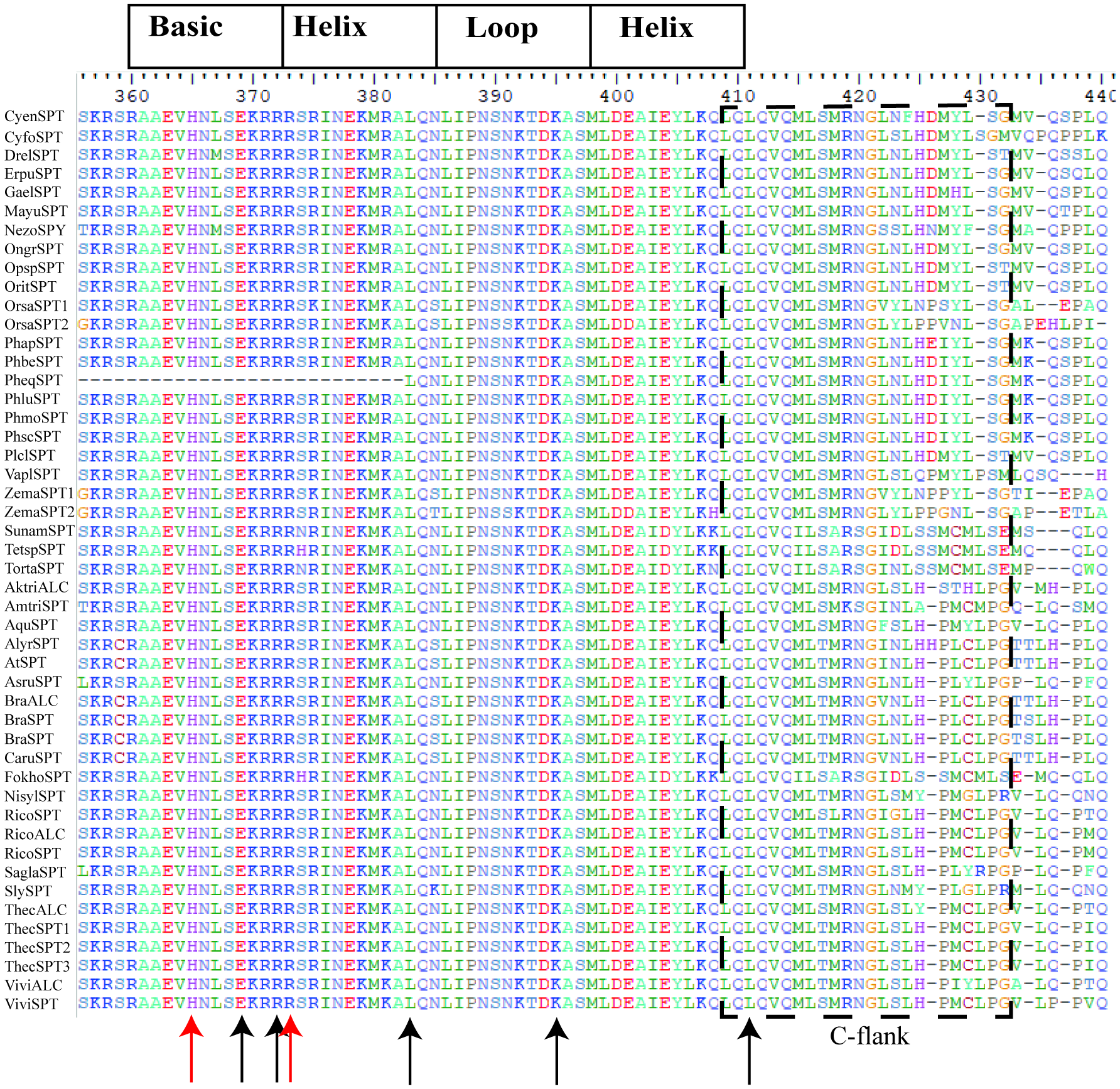

Supplement: Figure S6 — Alignment of the bHLH domain of SPATULA/ALCATRAZ proteins based on Pabon-Mora et al. (2014) extended with orchid gene lineages. The bHLH was drawn based on Toledo-Ortiz et al. (2003) and corresponds with positions K359-Q410. Within the bHLH domain, black arrows indicate positions E13, R16, L27, K39, L56, which are conserved in all bHLH plant and animal genes. The H9 and R17 positions (red arrows) show amino acids that provide the SPT/ALC proteins with G-box (CACGTG) binding activity. Black dashed boxes: N-flank and C-flank showing the conserved motif LQLQVQ. [file Image_6.TIF]

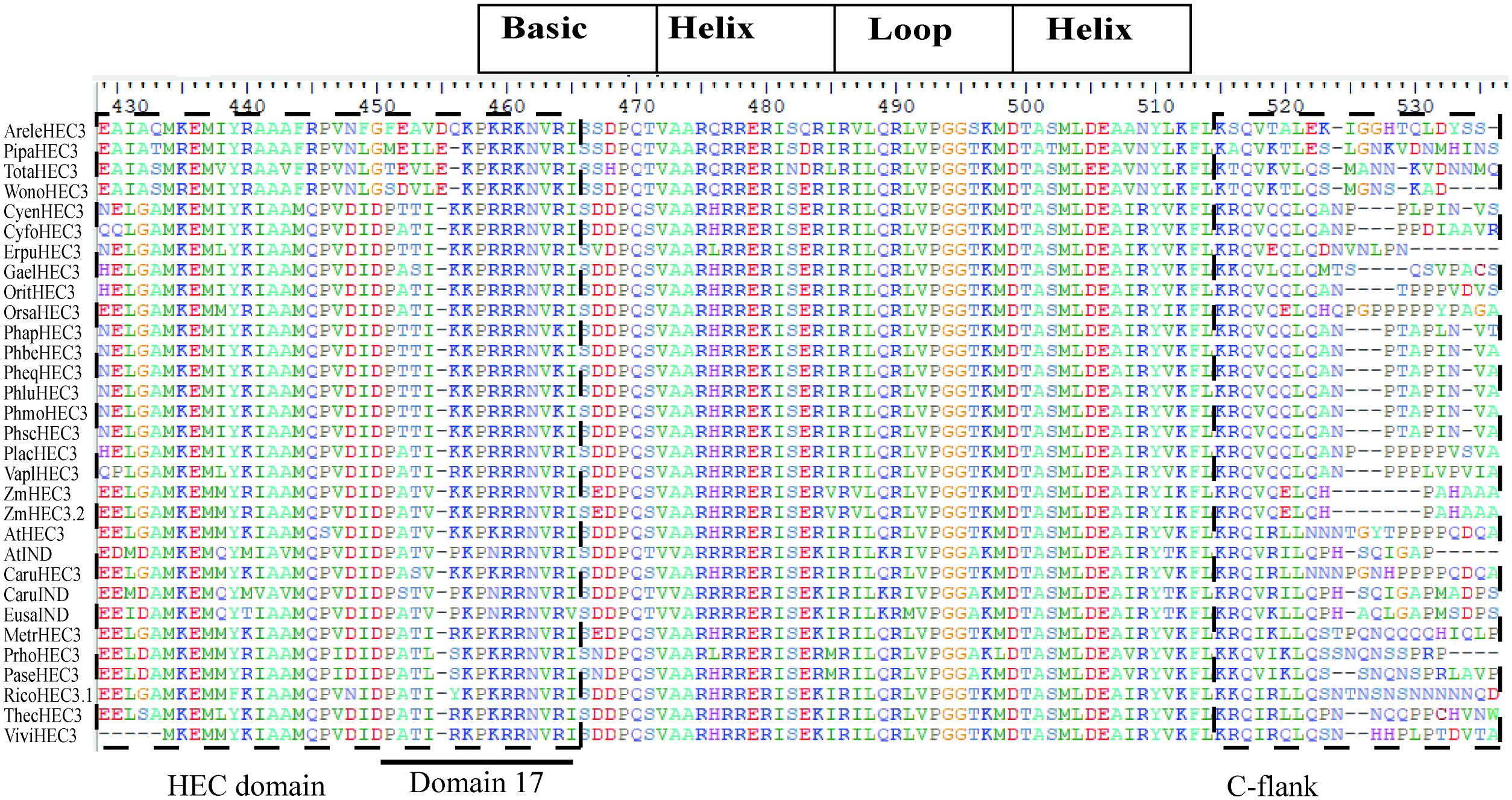

Supplement: Figure S7 — Alignment of the bHLH domain of HECATE3/INDEHISCENT proteins based on Pabon-Mora et al. (2014) extended with orchid gene lineages. The bHLH was drawn based on Toledo-Ortiz et al. (2003) and corresponds with positions N462-L515. Left black dashed box: N-flank of the bHLH domain: HEC domain (Kay et al., 2013) including domain 17 (Pires and Dolan, 2010). Right black dashed box: poorly conserved C-flank. [file Image_7.TIF]

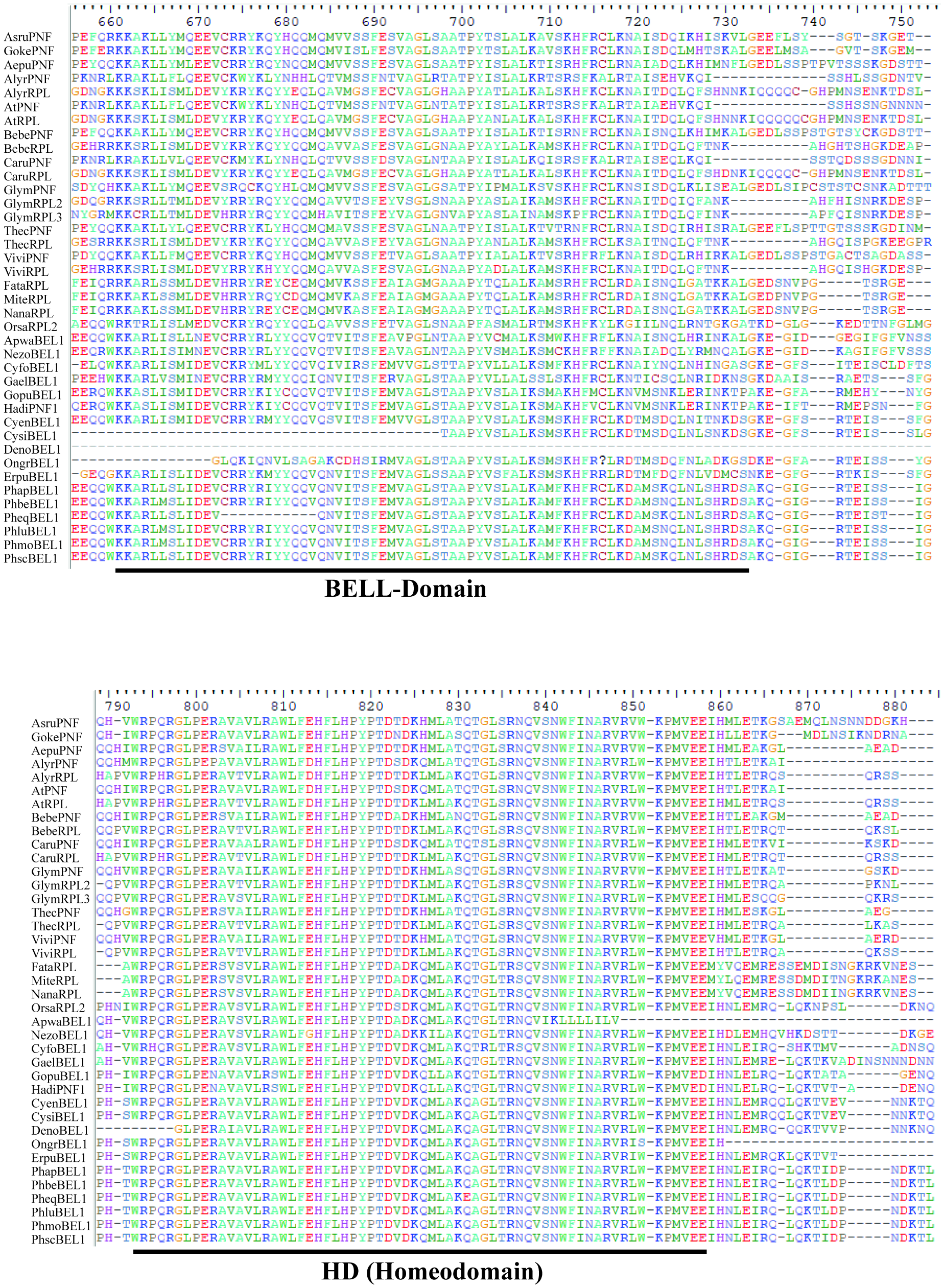

Supplement: Figure S8 — Alignment of the BELL-domain and the Homeo-domain of REPLUMLESS/POUNDFOOLISH (PNF) proteins based on Pabon-Mora et al. (2014) extended with orchid gene lineages. The BELL domain (Smith et al., 2002) and the conserved Homeodomain, based on Mukherjee et al. (2009) were drawn. [file Image_8.TIF]

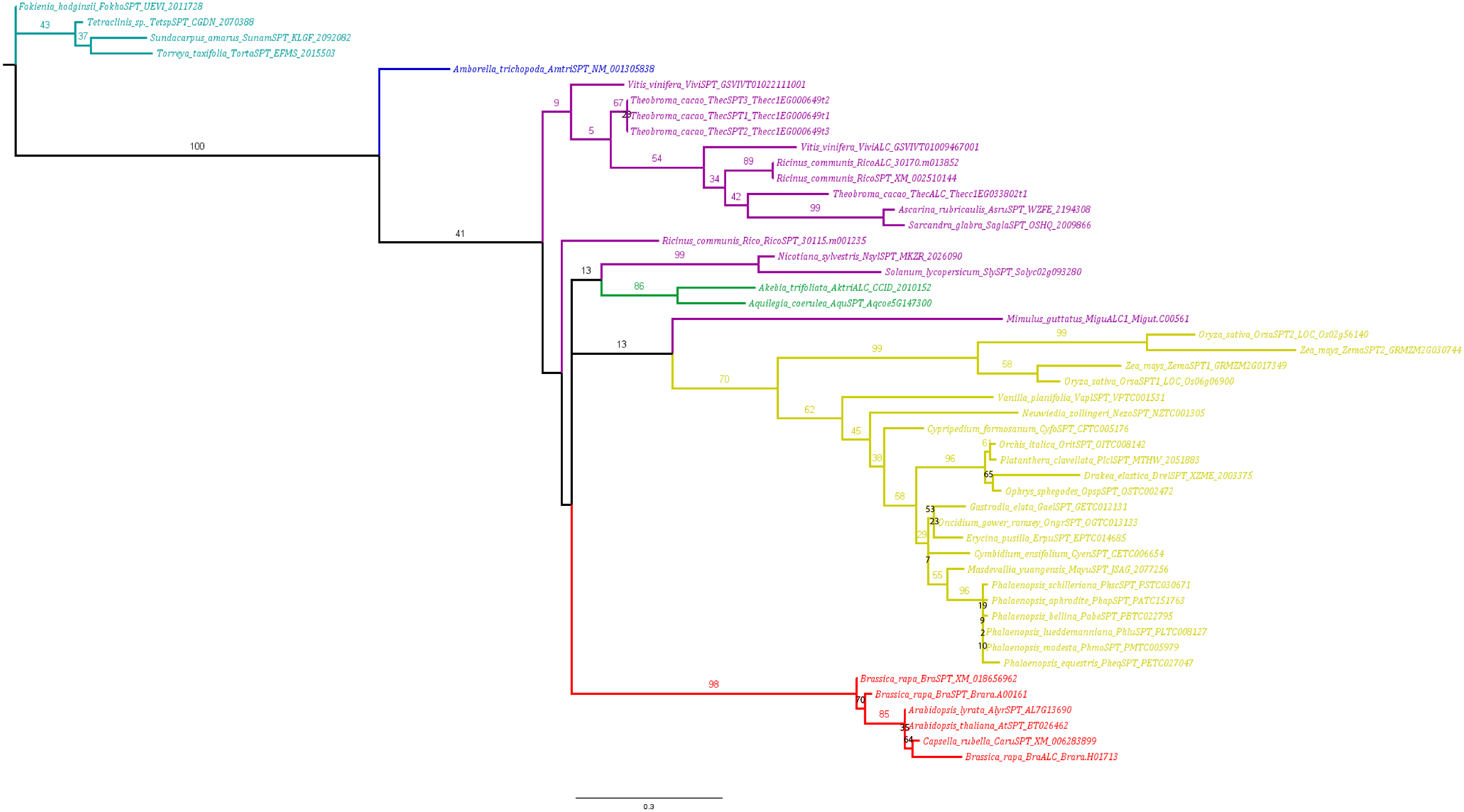

Supplement: Figure S9 — ML tree of the SPATULA/ALCATRAZ genes in seed plants. Branch colors denote the following taxa: Persian green, Gymnosperms; Blue, Basal angiosperms; Middle washed yellow, Monocots; Green, Basal eudicots; Purple, Core eudicots; Red, Brassicaceae. Bootstrap values are placed above the nodes. [file Image_9.TIF]

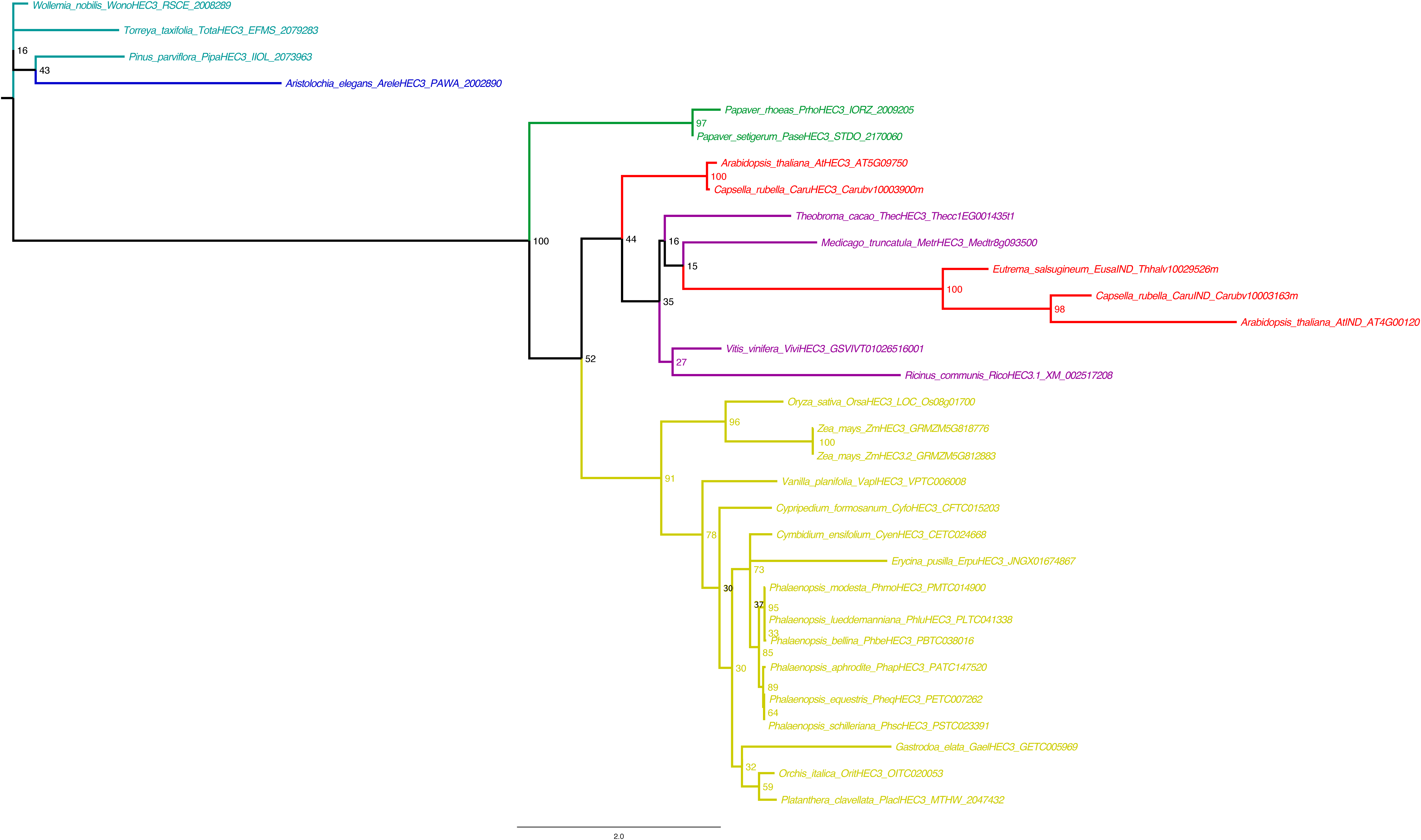

Supplement: Figure S10 — ML tree of the HECATE3/INDEHISCENT genes in seed plants. Branch colors denote the following taxa: Persian green, Gymnosperms; Blue, Basal angiosperms; Middle washed yellow, Monocots; Green, Basal eudicots; Purple, Core eudicots; Red, Brassicaceae. Bootstrap values are placed above the nodes. [file Image_10.TIF]

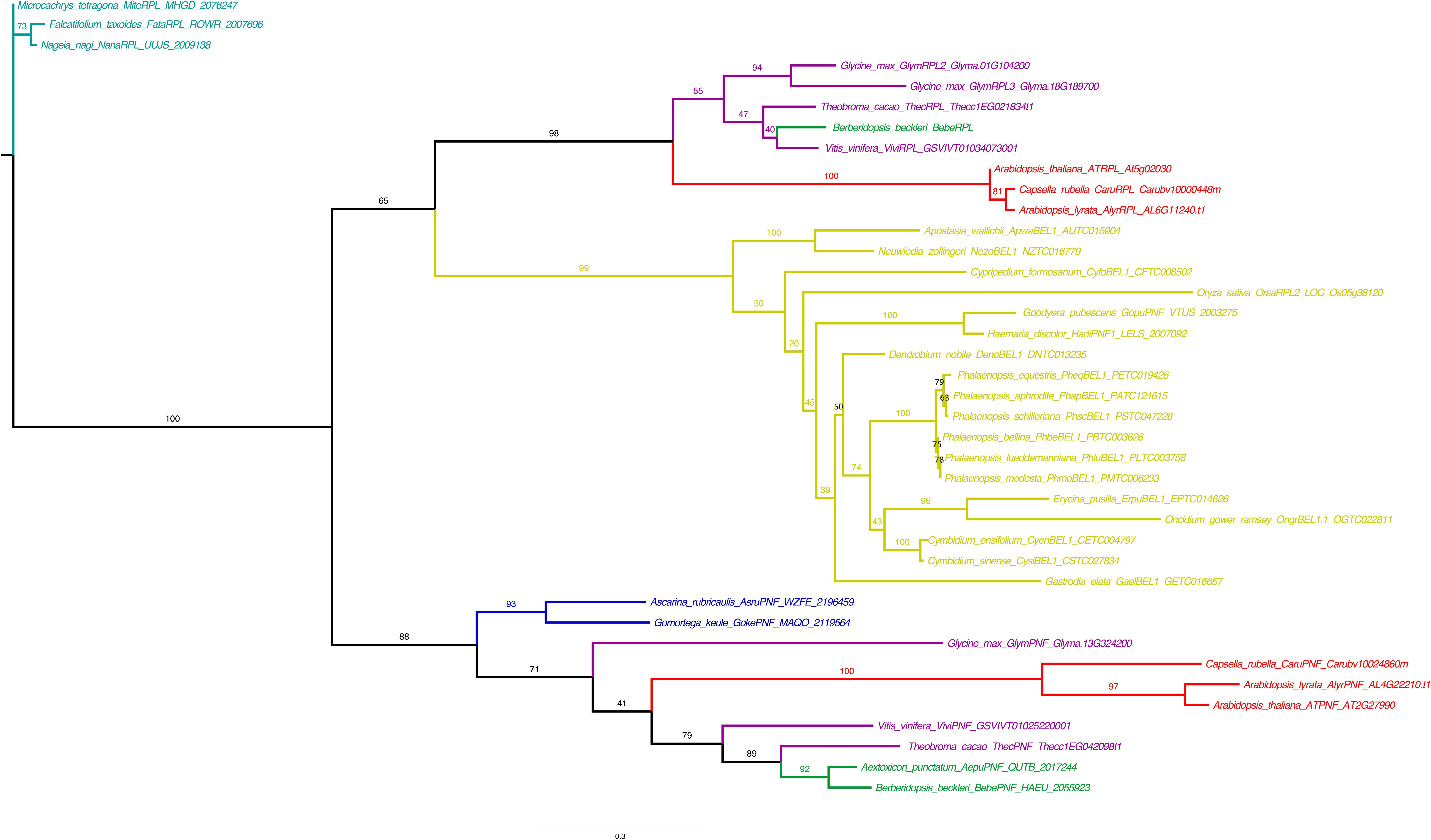

Supplement: Figure S11 — ML tree of the REPLUMLESS/POUNDFOOLISH genes in seed plants. Branch colors denote the following taxa: Persian green, Gymnosperms; Blue, Basal angiosperms; Middle washed yellow, Monocots; Green, Basal eudicots; Purple, Core eudicots; Red, Brassicaceae. Bootstrap values are placed above the nodes. [file Image_11.TIF]
