# Supplementary material for: Morphological and Molecular Characterization of Orchid Fruit Development
Source: Front Plant Sci. 2019 Feb 19;10:137. doi: 10.3389/fpls.2019.00137 (PMC6390509; doi:10.3389/fpls.2019.00137)
Supplement: Table S1 — AttB-primers used for the creation of inserts for Gateway cloning. [file Data_Sheet_1.docx]

Supplementary Material

**Morphological and molecular characterization of orchid fruit development**

**Anita Dirks-Mulder, Israa Ahmed, Mark uit het Broek, Louie Krol, Nino Menger, Jasmijn Snier, Anne van Winzum, Anneke de Wolf, Martijn van ’t Wout, Jamie J. Zeegers, Roland Butôt, Reinout Heijungs, Bertie Joan van Heuven, Jaco Kruizinga, Rob Langelaan, Erik F. Smets, Wim Star, Marian Bemer & Barbara Gravendeel^*^**

***Correspondence**:
Barbara Gravendeel
email: [Barbara.Gravendeel@naturalis.nl](mailto:Barbara.Gravendeel@naturalis.nl)

Table S1: AttB-primers used for the creation of inserts for Gateway cloning.

| **Primer Name** | **Sequence** | **Specific part** | **T_m_ specific part (°C)** |
| --- | --- | --- | --- |
| EpMADS3_attB1_Y2H | GGGGACAAGTTTGTACAAAAAAGCAGGCTYYATGGGGAGAGGGAGAGTTGAA | ATGGGGAGAGGGAGAGTTGAA | 61.2 |
| EpMADS3_attB2_Y2H | GGGGACCACTTTGTACAAGAAAGCTGGGTYCTAAAGCATCCACCCAAGCATAA | CTAAAGCATCCACCCAAGCATAA | 60.9 |
| EpMADS8_attB1_Y2H | GGGGACAAGTTTGTACAAAAAAGCAGGCTYYATGGGAAGAGGGAGAGTTGAACT | ATGGGAAGAGGGAGAGTTGAACT | 62.9 |
| EpMADS8_attB2_Y2H | GGGGACCACTTTGTACAAGAAAGCTGGGTYTCATGCAAGCCATCCTGGT | TCATGCAAGCCATCCTGGT | 57.5 |
| EpMADS9_attB1_Y2H | GGGGACAAGTTTGTACAAAAAAGCAGGCTYYATGGGTAGAGGGAGAGTGGAACT | ATGGGTAGAGGGAGAGTGGAACT | 64.6 |
| EpMADS9_attB2_Y2H | GGGGACCACTTTGTACAAGAAAGCTGGGTYCTACTCGTAGGAGCCAGAAATTTGTC | CTACTCGTAGGAGCCAGAAATTTGTC | 66.2 |
| EpMADS10_attB1_Y2H | GGGGACAAGTTTGTACAAAAAAGCAGGCTYYATGAGGAAGGGAAAGGTGCAAC | ATGAGGAAGGGAAAGGTGCAAC | 62.1 |
| EpMADS10_attB2_Y2H | GGGGACCACTTTGTACAAGAAAGCTGGGTYTCAGACACCTGAGGGACACTTTTG | TCAGACACCTGAGGGACACTTTTG | 65.2 |
| EpMADS11_attB1_Y2H | GGGGACAAGTTTGTACAAAAAAGCAGGCTYYATGGGAAGGGGGAGAGTTCA | ATGGGAAGGGGGAGAGTTCA | 60.5 |
| EpMADS11_attB2_Y2H | GGGGACCACTTTGTACAAGAAAGCTGGGTYTTAGGTTGGAGAGCGAAGCATC | TTAGGTTGGAGAGCGAAGCATC | 62.1 |
| EpMADS12_attB1_Y2H | GGGGACAAGTTTGTACAAAAAAGCAGGCTYYATGGGAAGAGGAAGAGTTCAGTTAAA | ATGGGAAGAGGAAGAGTTCAGTTAAA | 62.9 |
| EpMADS12_attB2_Y2H | GGGGACCACTTTGTACAAGAAAGCTGGGTYCTATCCTTTCAGATGGTTGAGCATC | CTATCCTTTCAGATGGTTGAGCATC | 64.1 |
| EpMADS14_attB1_Y2H | GGGGACAAGTTTGTACAAAAAAGCAGGCTYYATGGGGAGAGGAAAGTTAGAGAT | ATGGGGAGAGGAAAGTTAGAGAT | 60.9 |
| EpMADS14_attB2_Y2H | GGGGACCACTTTGTACAAGAAAGCTGGGTYTTATAAGCAATGTGCTAAACTCTGATT | TTATAAGCAATGTGCTAAACTCTGATT | 60.8 |
| EpMADS15_attB1_Y2H | GGGGACAAGTTTGTACAAAAAAGCAGGCTYYATGGGGAGGGGGAAAATAGAG | ATGGGGAGGGGGAAAATAGAG | 61.2 |
| EpMADS15_attB2_Y2H | GGGGACCACTTTGTACAAGAAAGCTGGGTYTTAAGATAGACTAAGATCATGAGATTCATAACCC | TTAAGATAGACTAAGATCATGAGATTCATAACCC | 67.9 |
| EpMADS18_attB1_Y2H | GGGGACAAGTTTGTACAAAAAAGCAGGCTYYATGGCGCGGGAGAAAATAAAG | ATGGCGCGGGAGAAAATAAAG | 59.5 |
| EpMADS18_attB2_Y2H | GGGGACCACTTTGTACAAGAAAGCTGGGTYTCATTTCCAGCCTGAGCATG | TCATTTCCAGCCTGAGCATG | 58.4 |
| EpMADS20_attB1_Y2H | GGGGACAAGTTTGTACAAAAAAGCAGGCTYYATGGAGAAGATTAACATGAAGGGAAGG | ATGGAGAAGATTAACATGAAGGGAAGG | 65.3 |
| EpMADS20_attB2_Y2H | GGGGACCACTTTGTACAAGAAAGCTGGGTYTTAGTTGGATGGTAGGCTATTTCCTG | TTAGTTGGATGGTAGGCTATTTCCTG | 64.6 |
| EpMADS21_attB1_Y2H | GGGGACAAGTTTGTACAAAAAAGCAGGCTYYATGGAGCCCAAGGAGAAGATG | ATGGAGCCCAAGGAGAAGATG | 61.2 |
| EpMADS21_attB2_Y2H | GGGGACCACTTTGTACAAGAAAGCTGGGTYTTACCCAAGCTGCAAAGTAGTCTG | TTACCCAAGCTGCAAAGTAGTCTG | 63.6 |
| EpMADS22_attB1_Y2H | GGGGACAAGTTTGTACAAAAAAGCAGGCTYYATGATGGAGCCAAAGGAAAAGATG | ATGATGGAGCCAAAGGAAAAGATG | 62 |
| EpMADS22_attB2_Y2H | GGGGACCACTTTGTACAAGAAAGCTGGGTYTTACCCTAATTGTAGGGCAGTTTGTT | TTACCCTAATTGTAGGGCAGTTTGTT | 62.9 |
| EpMADS23_attB1_Y2H | GGGGACAAGTTTGTACAAAAAAGCAGGCTYYATGGGAAGGGGCAAAATTGAG | ATGGGAAGGGGCAAAATTGAG | 59.5 |
| EpMADS23_attB2_Y2H | GGGGACCACTTTGTACAAGAAAGCTGGGTYTTATGTAGAATGATGTGCTTTTGATTC | TTATGTAGAATGATGTGCTTTTGATTC | 60.8 |

Table S2: Number of cell layers of *Erycina pusilla*, *Epipactis helleborine* and *Cynorkis fastigiata* fruits during fruit development. Measurements were performed. Abbreviations: DAP = days after pollination, WAP = weeks after pollination, Cf = *Cynorkis fastigiata*, Eh = *Epipactis helleborine*.

| ***Erycina pusilla*** | **Number of cell layers** | | | | ***Cynorkis fastigiata*** | **Number of cell layers** | | | | | ***Epipactis helleborine*** | **Number of cell layers** | | | |
| --- | --- | --- | --- | --- | --- | --- | --- | --- | --- | --- | --- | --- | --- | --- | --- |
| **Slide** | **DAP0** |  |  |  | **Slide** | **Cf01.2** | **Indehisced fruit** |  |  |  | **Slide** | **Eh011.2** | **Indehisced fruit** |  |  |
| 1 | 17 | 17 | 17 | 16 | 1 | 8 | 8 | 6 | 6 | 7 | 1 | 8 | 10 | 8 | 9 |
| 2 | 18 | 18 | 19 | 17 | 2 | 6 | 7 | 7 | 7 | 7 | 2 | 8 | 8 | 9 | 8 |
| 3 | 18 | 18 | 17 | 19 | 3 | 7 | 7 | 6 | 7 | 7 | 3 | 10 | 9 | 8 | 9 |
| **Slide** | **DAP7** |  |  |  | **Slide** | **Cf020.3** | **Indehisced fruit** |  |  |  | 4 | 9 | 8 | 8 | 8 |
| 1 | 17 | 14 | 14 | 16 | 1 | 7 | 6 | 7 | 8 | 7 | **Slide** | **Eh021.2** | **Indehisced fruit** |  |  |
| 2 | 13 | 13 | 14 | 14 | 2 | 6 | 7 | 7 | 8 | 6 | 1 | 9 | 9 | 9 | 10 |
| 3 | 13 | 14 | 15 | 14 | 3 | 6 | 6 | 8 | 7 | 7 | 2 | 10 | 10 | 10 | 9 |
| **Slide** | **WAP2** |  |  |  | **Slide** | **Cf041.3** | **Indehisced fruit** |  |  |  | 3 | 9 | 10 | 10 | 8 |
| 1 | 15 | 18 | 16 | 17 | 1 | 7 | 6 | 6 | 6 |  | **Slide** | **Eh033.2** | **Indehisced fruit** |  |  |
| 2 | 16 | 17 | 17 | 18 | 2 | 7 | 7 | 7 | 7 |  | 1 | 7 | 8 | 8 | 7 |
| 3 | 17 | 19 | 16 | 19 | 3 | 8 | 7 | 7 | 6 |  | 2 | 8 | 7 | 8 | 7 |
| **Slide** | **WAP7** |  |  |  | **Slide** | **Cf051.3** | **Indehisced fruit** |  |  |  | 3 | 7 | 7 | 8 | 8 |
| 1 | 13 | 14 | 14 | 14 | 1 | 7 | 6 | 6 | 7 |  | 4 | 8 | 8 | 7 | 8 |
| 2 | 15 | 13 | 14 | 14 | 2 | 8 | 8 | 7 | 6 |  | **Slide** | **Eh053.1** | **Indehisced fruit** |  |  |
| 3 | 14 | 15 | 13 | 13 | 3 | 7 | 7 | 7 | 6 |  | 1 | 10 | 10 | 8 | 9 |
| **Slide** | **WAP15** |  |  |  | **Slide** | **Cf-M16** | **Dehisced fruit** |  |  |  | 2 | 10 | 9 | 10 | 11 |
| 1 | 16 | 16 | 15 | 17 | 1 | 9 | 9 | 9 | 8 |  | 3 | 8 | 9 | 8 | 9 |
| 2 | 15 | 17 | 16 | 15 | 2 | 9 | 8 | 7 | 8 |  | **Slide** | **Eh063.2** | **Indehisced fruit** |  |  |
| **Slide** | **WAP16** |  |  |  | 3 | 9 | 7 | 8 | 8 |  | 1 | 9 | 10 | 9 | 9 |
| 1 | 15 | 15 | 15 | 14 |  |  |  |  |  |  | 2 | 8 | 9 | 10 | 10 |
| 2 | 15 | 14 | 16 | 16 |  |  |  |  |  |  | 3 | 10 | 9 | 10 | 11 |
| 3 | 14 | 14 | 13 | 14 |  |  |  |  |  |  | 4 | 9 | 8 | 10 | 9 |
|  |  |  |  |  |  |  |  |  |  |  | **Slide** | **Eh072.1** | **Dehisced fruit** |  |  |
|  |  |  |  |  |  |  |  |  |  |  | 1 | 8 | 8 | 7 | 8 |
|  |  |  |  |  |  |  |  |  |  |  | 2 | 7 | 8 | 8 | 8 |
|  |  |  |  |  |  |  |  |  |  |  | 3 | 9 | 8 | 7 | 8 |
|  |  |  |  |  |  |  |  |  |  |  | 4 | 7 | 8 | 8 | 8 |

Table S3: Transcript primer sequences and amplicon characteristics used for quantitative real-time PCR validation of the expression profiles of different transcripts, following MIQE guidelines (Bustin et al., 2009). The sequences listed here were downloaded from NCBI GenBank (www.ncbi.nlm.nih.gov), Orchidstra (orchidstra2.abrc.sinica.edu.tw) and our own fruit transcriptome dataset (in prep.).

| **Accession** | **Target**  **Gene** | **Transcription**  **factor** | **Primer Name** | **Sequence** | **Tm (°C)** | **GC (%)** | **Amplicon (bp)** |
| --- | --- | --- | --- | --- | --- | --- | --- |
| KJ002743 | SVP | MADS-box | EpMADS18-FW1 | GCAGAACTTCAGATGGTAGGAA | 64 | 45.5 | 111 |
|  |  |  | EpMADS18-RV1 | TCCGCAATGAGTCACATTAGTTAC | 65 | 41.7 |  |
| EPTC014685 | SPT | bHLH-like | EpSPT-FW1 | GGGACCTTCCTGCTTCTTTG | 64.8 | 55 | 101 |
|  |  |  | EpSPT-RV1 | CCTCCACATCCTCAATATCCAAC | 64.7 | 47.8 |  |
| -* | HEC3 | bHLH-like | EpHEC3-FW3 | CCTACGACCATCAAGAAGCC | 64.4 | 55 | 124 |
|  |  |  | EpHEC3-RV3 | GAACGAGTCTCTGAAGAATCCTAAT | 64.1 | 40 |  |
| EPTC014626 | RPL | TALE-like | EpRPL-FW2 | TCTTCCTCTAGTAACCAACATCTTCTA | 64.7 | 37 | 139 |
|  |  |  | EpRPL-RV1 | CTCCGTTCCCTCCGAGAT | 64.4 | 61.1 |  |

******ErpuHEC3-like* gene from our fruit transcriptome dataset: CACACCCAAAACGATCAAAACGAACTCGGCGCAATGAAGGAAATGCTATATAAGATCGCCGCCATGCAACCCGTCGACATCGACCCTACGACCATCAAGAAGCCGCGCCGTCGAAATGTACGAATAAGTGTCGACCCGCAGAGCGTCGCCGCGCGTCTCCGACGCGAGCGGATCAGCGAGAGAATTAGGATTCTTCAGAGACTCGTTCCCGGAGGCACTAAGATGGACACTGCATCTATGCTTGATGAAGCAATTAAGTATGTTAAATTTCTGAAGAGACAAGTGGAACAACTTCAAGATAATGTAAATTTGCCAAATAATATTGTTTCTTTAGCTGAATTTCGGTTTGGTTGTGATGGAAATGGA

Table S4: Difference in MADS-box gene expression between developmental stages of the fruit of *E. pusilla* as calculated using a variance analysis of measures using a Tukey multi-comparisons test.
P-value style: GP: >0.05 (ns), <0.05 (*), <0.01 (**), <0.001 (***), <0.0001 (****). No value = No expression. Abbreviations: DAP = days after pollination, WAP = weeks after pollination.

|  | ***EpMADS3*** | ***EpMADS8*** | ***EpMADS9*** | ***EpMADS10*** | ***EpMADS11*** | ***EpMADS12*** | ***EpMADS14*** | ***EpMADS15*** |
| --- | --- | --- | --- | --- | --- | --- | --- | --- |
| 0 DAP vs. 1 DAP | ns | ns | ns | ns | *** | **** | ns | ns |
| 0 DAP vs. 3 DAP | ns | ns | ns | *** | **** | **** | ** | ** |
| 0 DAP vs. 5 DAP | * | ns | ns | **** | **** | **** | **** | **** |
| 0 DAP vs. 9 DAP | ns | ns | ns | ns | ns | ns |  | ns |
| 0 DAP vs. 2 WAP | ns | ns | ns |  | ns | ns |  | ns |
| 0 DAP vs. 3 WAP | ns | ns | ns | ns | ns | ns | ns | ns |
| 0 DAP vs. 4 WAP | ns | ns | ns | ns | ns | ns | ns | ns |
| 0 DAP vs. 8 WAP | ns | ns | ns | ns | ns | ns | ns | ns |
| 0 DAP vs. 12 WAP | ns | ns | ns | ns | ns | ns | ns | ns |
| 0 DAP vs. 16 WAP | ns | **** | ns | ns | ns | ns | ns | ns |
| 0 DAP vs. SEED | ns | ns | * | ns | ** |  | *** |  |
| 1 DAP vs. 3 DAP | ns | ns | ns | ns | ns | ns | ns | ns |
| 1 DAP vs. 5 DAP | ns | ns | ns | **** | **** | **** | **** | **** |
| 1 DAP vs. 9 DAP | ns | ns | * | * | **** | **** |  | ns |
| 1 DAP vs. 2 WAP | ns | ns | ns |  | **** | **** |  | ns |
| 1 DAP vs. 3 WAP | ns | ns | ns | ** | *** | **** | ns | ns |
| 1 DAP vs. 4 WAP | ns | ns | ns | **** | **** | **** | ns | ns |
| 1 DAP vs. 8 WAP | ns | ns | ns | *** | **** | **** | ns | ns |
| 1 DAP vs. 12 WAP | ns | ns | ns | *** | **** | **** | ns | ns |
| 1 DAP vs. 16 WAP | ns | **** | ns | ** | **** | **** | ns | ns |
| 1 DAP vs. SEED | * | ns | *** | * | ns |  | ** |  |
| 3 DAP vs. 5 DAP | ns | ns | ns | **** | **** | **** | **** | **** |
| 3 DAP vs. 9 DAP | ns | ns | ns | *** | **** | **** |  | ** |
| 3 DAP vs. 2 WAP | ns | ns | ns |  | **** | **** |  | ** |
| 3 DAP vs. 3 WAP | ns | ns | ns | **** | **** | **** | ns | ** |
| 3 DAP vs. 4 WAP | ns | ns | ns | **** | **** | **** | *** | **** |
| 3 DAP vs. 8 WAP | ns | ns | ns | **** | **** | **** | ** | ** |
| 3 DAP vs. 12 WAP | ns | ns | ns | **** | **** | **** | ** | ** |
| 3 DAP vs. 16 WAP | ns | **** | ns | **** | **** | **** | ** | *** |
| 3 DAP vs. SEED | ns | ns | ns | **** | ns |  | ns |  |
| 5 DAP vs. 9 DAP | ns | ns | ns | **** | **** | **** |  | **** |
| 5 DAP vs. 2 WAP | ns | * | ns |  | **** | **** |  | **** |
| 5 DAP vs. 3 WAP | ns | ns | ns | **** | **** | **** | **** | **** |
| 5 DAP vs. 4 WAP | ns | ns | ns | **** | **** | **** | **** | **** |
| 5 DAP vs. 8 WAP | ns | ns | ns | **** | **** | **** | **** | **** |
| 5 DAP vs. 12 WAP | ns | ns | ns | **** | **** | **** | **** | **** |
| 5 DAP vs. 16 WAP | ns | **** | ns | **** | **** | **** | **** | **** |
| 5 DAP vs. SEED | *** | * | ns | **** | **** |  | **** |  |
| 9 DAP vs. 2 WAP | ns | ns | ** |  | ns | ns |  | ns |
| 9 DAP vs. 3 WAP | ns | ns | * | ns | ns | ns |  | ns |
| 9 DAP vs. 4 WAP | ns | ns | ns | ns | ns | ns |  | ns |
| 9 DAP vs. 8 WAP | ns | ns | ns | ns | ns | ns |  | ns |
| 9 DAP vs. 12 WAP | ns | ns | ns | ns | ns | ns |  | ns |
| 9 DAP vs. 16 WAP | ns | **** | ns | ns | ns | ns |  | ns |
| 9 DAP vs. SEED | ns | ns | ns | ns | ** |  |  |  |
| 2 WAP vs. 3 WAP | ns | ns | ns |  | ns | ns |  | ns |
| 2 WAP vs. 4 WAP | ns | ns | ns |  | ns | ns |  | ns |
| 2 WAP vs. 8 WAP | ns | ns | ns |  | ns | ns |  | ns |
| 2 WAP vs. 12 WAP | ns | ns | ns |  | ns | ns |  | ns |
| 2 WAP vs. 16 WAP | ns | **** | ns |  | ns | ns |  | ns |
| 2 WAP vs. SEED | ns | ns | **** |  | ** |  |  |  |
| 3 WAP vs. 4 WAP | ns | ns | ns | ns | ns | ns | ns | ns |
| 3 WAP vs. 8 WAP | ns | ns | ns | ns | ns | ns | ns | ns |
| 3 WAP vs. 12 WAP | ns | ns | ns | ns | ns | ns | ns | ns |
| 3 WAP vs. 16 WAP | ns | **** | ns | ns | ns | ns | ns | ns |
| 3 WAP vs. SEED | ns | ns | ** | ns | * |  | * |  |
| 4 WAP vs. 8 WAP | ns | ns | ns | ns | ns | ns | ns | ns |
| 4 WAP vs. 12 WAP | ns | ns | ns | ns | ns | ns | ns | ns |
| 4 WAP vs. 16 WAP | ns | **** | ns | ns | ns | ns | ns | ns |
| 4 WAP vs. SEED | ns | ns | ns | ns | *** |  | **** |  |
| 8 WAP vs. 12 WAP | ns | ns | ns | ns | ns | ns | ns | ns |
| 8 WAP vs. 16 WAP | ns | **** | ns | ns | ns | ns | ns | ns |
| 8 WAP vs. SEED | ns | ns | ns | ns | *** |  | **** |  |
| 12 WAP vs. 16 WAP | ns | **** | ns | ns | ns | ns | ns | ns |
| 12 WAP vs. SEED | ns | ns | ns | ns | ** |  | **** |  |
| 16 WAP vs. SEED | ns | **** | ns | ns | *** |  | **** |  |

Table S4 continued: Difference in MADS-box gene expression between developmental stages of the fruit of *E. pusilla* as calculated using a variance analysis of measures using a Tukey multi-comparisons test.
P-value style: GP: >0.05 (ns), <0.05 (*), <0.01 (**), <0.001 (***), <0.0001 (****). No value = No expression. Abbreviations: DAP = days after pollination, WAP = weeks after pollination.

|  | ***EpMADS15*** | ***EpMADS18*** | ***EpMADS20*** | ***EpMADS21*** | ***EpMADS22*** | ***EpMADS23*** | ***EpHEC*** | ***EpRPL*** | ***EpSPT*** |
| --- | --- | --- | --- | --- | --- | --- | --- | --- | --- |
| 0 DAP vs. 1 DAP | ns | ns | ns | ns | ** | *** | ns | *** | ns |
| 0 DAP vs. 3 DAP | ** | ns | **** | ns | **** | **** | ns | ns | ns |
| 0 DAP vs. 5 DAP | **** | ns | **** | **** | **** | **** | ns | **** | ns |
| 0 DAP vs. 9 DAP | ns | ns | ns | ns | ns | ns | ns | ns | ** |
| 0 DAP vs. 2 WAP | ns | ns | ns | ns | ns | ns | ns | ns | **** |
| 0 DAP vs. 3 WAP | ns | ns | ns | ns | ns | *** | ns | ns | ** |
| 0 DAP vs. 4 WAP | ns | ns | ns | ns | ns | ns | ns | ns | ** |
| 0 DAP vs. 8 WAP | ns | ns | ns | ns | ns | ns | ns | ns | ** |
| 0 DAP vs. 12 WAP | ns | ns | ns | ns | ns | ns | ns | ns | *** |
| 0 DAP vs. 16 WAP | ns | **** | ns | ns | ns | ns | **** | **** | ns |
| 0 DAP vs. SEED |  | ns | ns | ns | **** | ** | ns | ns | **** |
| 1 DAP vs. 3 DAP | ns | ns | *** | ns | * | ns | ns | ns | ns |
| 1 DAP vs. 5 DAP | **** | ns | **** | **** | **** | **** | ns | ns | ns |
| 1 DAP vs. 9 DAP | ns | ns | ns | ns | ns | ns | ns | **** | ns |
| 1 DAP vs. 2 WAP | ns | ns | ns | ns | * | ns | ns | **** | ** |
| 1 DAP vs. 3 WAP | ns | ns | ns | ns | ns | ns | ns | **** | ns |
| 1 DAP vs. 4 WAP | ns | ns | ns | ns | ns | ns | ns | **** | ns |
| 1 DAP vs. 8 WAP | ns | ns | ns | ns | ns | ** | ns | ** | ns |
| 1 DAP vs. 12 WAP | ns | ns | ns | ns | * | ** | ns | ns | * |
| 1 DAP vs. 16 WAP | ns | **** | ns | ns | ** | ** | **** | **** | ns |
| 1 DAP vs. SEED |  | ns | ns | ns | * | ns | ns | ns | *** |
| 3 DAP vs. 5 DAP | **** | * | **** | **** | **** | **** | ns | ns | ns |
| 3 DAP vs. 9 DAP | ** | ns | **** | ns | **** | *** | ns | ** | **** |
| 3 DAP vs. 2 WAP | ** | ns | **** | ns | **** | *** | ns | ** | **** |
| 3 DAP vs. 3 WAP | ** | ns | **** | ns | **** | ns | ns | ** | **** |
| 3 DAP vs. 4 WAP | **** | ns | **** | ns | **** | **** | ns | * | **** |
| 3 DAP vs. 8 WAP | ** | ns | **** | ns | **** | **** | ns | ns | **** |
| 3 DAP vs. 12 WAP | ** | ns | **** | ns | **** | **** | ns | ns | **** |
| 3 DAP vs. 16 WAP | *** | **** | **** | ns | **** | **** | **** | **** | ns |
| 3 DAP vs. SEED |  | ns | **** | ns | ns | ns | ns | ns | **** |
| 5 DAP vs. 9 DAP | **** | ** | **** | **** | **** | **** | ns | **** | ** |
| 5 DAP vs. 2 WAP | **** | * | **** | **** | **** | **** | ns | **** | *** |
| 5 DAP vs. 3 WAP | **** | ** | **** | ** | **** | **** | ns | **** | ** |
| 5 DAP vs. 4 WAP | **** | * | **** | ** | **** | **** | ns | **** | * |
| 5 DAP vs. 8 WAP | **** | ns | **** | **** | **** | **** | ns | *** | * |
| 5 DAP vs. 12 WAP | **** | ns | **** | **** | **** | **** | ns | ns | *** |
| 5 DAP vs. 16 WAP | **** | *** | **** | **** | **** | **** | **** | **** | ns |
| 5 DAP vs. SEED |  | ns | **** | **** | **** | **** | ns | ns | **** |
| 9 DAP vs. 2 WAP | ns | ns | ns | ns | ns | ns | ns | ns | ns |
| 9 DAP vs. 3 WAP | ns | ns | ns | ns | ns | ns | ns | ns | ns |
| 9 DAP vs. 4 WAP | ns | ns | ns | ns | ns | ns | ns | ns | ns |
| 9 DAP vs. 8 WAP | ns | ns | ns | ns | ns | ns | ns | ns | ns |
| 9 DAP vs. 12 WAP | ns | ns | ns | ns | ns | ns | ns | ** | ns |
| 9 DAP vs. 16 WAP | ns | **** | ns | ns | ns | ns | **** | **** | * |
| 9 DAP vs. SEED |  | ns | ns | ns | **** | ns | ns | * | ns |
| 2 WAP vs. 3 WAP | ns | ns | ns | ns | ns | ns | ns | ns | ns |
| 2 WAP vs. 4 WAP | ns | ns | ns | ns | ns | ns | ns | ns | ns |
| 2 WAP vs. 8 WAP | ns | ns | ns | ns | ns | ns | ns | ns | ns |
| 2 WAP vs. 12 WAP | ns | ns | ns | ns | ns | ns | ns | ** | ns |
| 2 WAP vs. 16 WAP | ns | **** | ns | ns | ns | ns | **** | **** | ** |
| 2 WAP vs. SEED |  | ns | ns | ns | **** | ns | ns | * | ns |
| 3 WAP vs. 4 WAP | ns | ns | ns | ns | ns | * | ns | ns | ns |
| 3 WAP vs. 8 WAP | ns | ns | ns | ns | ns | ** | ns | ns | ns |
| 3 WAP vs. 12 WAP | ns | ns | ns | ns | ns | ** | ns | ** | ns |
| 3 WAP vs. 16 WAP | ns | **** | ns | ns | ns | *** | **** | **** | * |
| 3 WAP vs. SEED |  | ns | ns | ns | *** | ns | ns | ns | ns |
| 4 WAP vs. 8 WAP | ns | ns | ns | * | ns | ns | ns | ns | ns |
| 4 WAP vs. 12 WAP | ns | ns | ns | ns | ns | ns | ns | * | ns |
| 4 WAP vs. 16 WAP | ns | **** | ns | * | ns | ns | **** | **** | ns |
| 4 WAP vs. SEED |  | ns | ns | ns | **** | ns | ns | ns | ns |
| 8 WAP vs. 12 WAP | ns | ns | ns | ns | ns | ns | ns | ns | ns |
| 8 WAP vs. 16 WAP | ns | **** | ns | ns | ns | ns | **** | **** | ns |
| 8 WAP vs. SEED |  | ns | ns | ns | *** | * | ns | ns | ns |
| 12 WAP vs. 16 WAP | ns | **** | ns | ns | ns | ns | **** | **** | ** |
| 12 WAP vs. SEED |  | ns | ns | ns | **** | * | ns | ns | ns |
| 16 WAP vs. SEED |  | **** | ns | ns | **** | * | **** | **** | **** |

Sequences for Table S5, S6 and S7 were retrieved from NCBI Genbank (www.ncbi.nlm.nih.gov), OneKP (https://sites.google.com/a/ualberta.ca/onekp), Phytozome (https://phytozome.jgi.doe.gov) and Orchidstra (orchidstra2.abrc.sinica.edu.tw).

Table S5: Accession numbers of *SPTATULA/ALCATRAZ* *bHLH* transcription factors sequences used in the alignment. The Orchidaceae subfamilies are in parentheses.

| **Gene name** | **Species** | **Family** | **Accession number** | **Database** |
| --- | --- | --- | --- | --- |
| **Gymnosperms** | | | | |
| *FokhoSPT* | *Fokienia hodginsii* | Cupressaceae | UEVI-2011728 | OneKP |
| *TetspSPT* | *Tetraclinis sp.* | Cupressaceae | CGDN-2070388 | OneKP |
| *SunamSPT* | *Sundacarpus amarus* | Podocarpaceae | KLGF-2092082 | OneKP |
| *TortaSPT* | *Torreya taxifolia* | Taxaceae | EFMS-2015503 | OneKP |
| **Basal Angiosperms** | | | | |
| *AmtriSPT* | *Amborella trichopoda* | Amborellaceae | NM_001305838 | Genbank |
| *AsruSPT* | *Ascarina rubricaulis* | Chloranthaceae | WZFE-2194308 | OneKP |
| *SaglaSPT* | *Sarcandra glabra* | Chloranthaceae | OSHQ-2009866 | OneKP |
| **Monocots** | | | | |
| *NezoSPT* | *Neuwiedia zollingeri* | Orchidaceae (Apostasioideae) | NZTC001305 | Orchidstra |
| *CyfoSPT* | *Cypripedium formosanum* | Orchidaceae (Cypripedioideae) | CFTC005176 | Orchidstra |
| *CyenSPT* | *Cymbidium ensifolium* | Orchidaceae (Epidendroideae) | CETC006654 | Orchidstra |
| *ErpuSPT* | *Erycina pusilla* | Orchidaceae (Epidendroideae) | EPTC014685 | Orchidstra |
| *GaelSPT* | *Gastrodia elata* | Orchidaceae (Epidendroideae) | GETC012131 | Orchidstra |
| *MayuSPT* | *Masdevallia yuangensis* | Orchidaceae (Epidendroideae) | JSAG-2077256 | OneKP |
| *OngrSPT* | *Oncidium gower ramsey* | Orchidaceae (Epidendroideae) | OGTC013133 | Orchidstra |
| *PhapSPT* | *Phalaenopsis aphrodite* | Orchidaceae (Epidendroideae) | PATC151763 | Orchidstra |
| *PhbeSPT* | *Phalaenopsis bellina* | Orchidaceae (Epidendroideae) | PBTC022795 | Orchidstra |
| *PheqSPT* | *Phalaenopsis equestris* | Orchidaceae (Epidendroideae) | PETC027047 | Orchidstra |
| *PhluSPT* | *Phalaenopsis lueddemanniana* | Orchidaceae (Epidendroideae) | PLTC008127 | Orchidstra |
| *PhmoSPT* | *Phalaenopsis modesta* | Orchidaceae (Epidendroideae) | PMTC005979 | Orchidstra |
| *PhscSPT* | *Phalaenopsis schilleriana* | Orchidaceae (Epidendroideae) | PSTC030671 | Orchidstra |
| *DrelSPT* | *Drakea elastica* | Orchidaceae (Orchidoideae) | XZME-2003375 | OneKP |
| *OritSPT* | *Orchis italica* | Orchidaceae (Orchidoideae) | OITC008142 | Orchidstra |
| *OpspSPT* | *Ophrys sphegodes* | Orchidaceae (Orchidoideae) | OSTC002472 | Orchidstra |
| *PlclSPT* | *Platanthera clavellata* | Orchidaceae (Orchidoideae) | MTHW-2051883 | OneKP |
| *VaplSPT* | *Vanilla planifolia* | Orchidaceae (Vanilloideae) | VPTC001531 | Orchidstra |
| *OrsaSPT1* | *Oryza sativa* | Poaceae | LOC-Os06g06900 | Phytozome |
| *OrsaSPT2* | *Oryza sativa* | Poaceae | LOC-Os02g56140 | Phytozome |
| *ZemaSPT1* | *Zea mays* | Poaceae | GRMZM2G017349 | Phytozome |
| *ZemaSPT2* | *Zea mays* | Poaceae | GRMZM2G030744 | Phytozome |
| **Basal Eudicots** | | | | |
| *AktriALC* | *Akebia trifoliata* | Lardizabalaceae | CCID-2010152 | OneKP |
| *AquSPT* | *Aquilegia coerulea* | Ranunculaceae | Aqcoe5G147300 | Phytozome |
| **Core Eudicots** | | | | |
| *AlyrSPT* | *Arabidopsis lyrata* | Brassicaceae | AL7G13690 | Phytozome |
| *AtSPT* | *Arabidopsis thaliana* | Brassicaceae | BT026462 (AT4G36930) | Genbank |
| *BraALC* | *Brassica rapa* | Brassicaceae | Brara.H01713 | Phytozome |
| *BraSPT* | *Brassica rapa* | Brassicaceae | XM_018656962 Brara.A00161 | Genbank Phytozome |
| *CaruSPT* | *Capsella rubella* | Brassicaceae | XM_006283899 | Genbank |
| *RicoSPT* | *Ricinus communis* | Euphorbiaceae | XM_002510144 30115.M001235 | Genbank Phytozome |
| *RicoALC* | *Ricinus communis* | Euphorbiaceae | 30170.m013852  (30170.t000264) | Phytozome |
| *NsylSPT* | *Nicotiana sylvestris* | Solanaceae | MKZR-2026090 | OneKP |
| *SlySPT* | *Solanum lycopersicum* | Solanaceae | Solyc02g093280 | Phytozome |
| *ThecALC* | *Theobroma cacao* | Sterculiaceae | Thecc1EG033802t.1 | Phytozome |
| *ThecSPT1* | *Theobroma cacao* | Sterculiaceae | Thecc1EG000649t.1 | Phytozome |
| *ThecSPT2* | *Theobroma cacao* | Sterculiaceae | Thecc1EG000649t3 | Phytozome |
| *ThecSPT3* | *Theobroma cacao* | Sterculiaceae | Thecc1EG000649t2 | Phytozome |
| *ViviALC* | *Vitis vinifera* | Vitaceae | GSVIVT01009467001 | Phytozome |
| *ViviSPT* | *Vitis vinifera* | Vitaceae | GSVIVG01022111001 | Phytozome |

Table S6: Accession numbers of *INDEHISCENT/HECASE3* sequences used in the alignments and phylogenetic analyses. The Orchidaceae subfamilies are in parentheses.

| **Gene name** | **Species** | **Family** | **Accession number** | **Database** |
| --- | --- | --- | --- | --- |
| **Gymnosperms** | | | | |
| *WonoHEC3* | *Wollemia nobilis* | Araucariaceae | RSCE-2008289 | oneKP |
| *PipaHEC3* | *Pinus parviflora* | Pinaceae | IIOL-2073963 | oneKP |
| *TotaHEC3* | *Torreya taxifolia* | Taxaceae | EFWS-2079283 | oneKP |
| **Basal Angiosperms** | | | | |
| *AreleHEC3* | *Aristolochia elegans* | Aristolochiaceae | PAWA-2002890 | oneKP |
| **Monocots** | | | | |
| *CyfoHEC3* | *Cypripedium formosanum* | Orchidaceae (Cypripedioideae) | CFTC015203 | Orchidstra |
| *CyenHEC3* | *Cymbidium ensifolium* | Orchidaceae (Epidendroideae) | CETC024668 | Orchidstra |
| *ErpuHEC3** | *Erycina pusilla* | Orchidaceae (Epidendroideae) | JNGX01674867 | - |
| *GaelHEC3* | *Gastrodoa elata* | Orchidaceae (Epidendroideae) | GETC005969 | Orchidstra |
| *PhapHEC3* | *Phalaenopsis aphrodite* | Orchidaceae (Epidendroideae) | PATC147520 | Orchidstra |
| *PhbeHEC3* | *Phalaenopsis bellina* | Orchidaceae (Epidendroideae) | PBTC038016 | Orchidstra |
| *PheqHEC3* | *Phalaenopsis equestris* | Orchidaceae (Epidendroideae) | PETC007262 | Orchidstra |
| *PhluHEC3* | *Phalaenopsis lueddemanniana* | Orchidaceae (Epidendroideae) | PLTC041338 | Orchidstra |
| *PhmoHEC3* | *Phalaenopsis modesta* | Orchidaceae (Epidendroideae) | PMTC014900 | Orchidstra |
| *PhscHEC3* | *Phalaenopsis schilleriana* | Orchidaceae (Epidendroideae) | PSTC023391 | Orchidstra |
| *OritHEC3* | *Orchis italica* | Orchidaceae (Orchidoideae) | OITC020053 | Orchidstra |
| *PlaclHEC3* | *Platanthera clavellata* | Orchidaceae (Orchidoideae) | MTHW-2047432 | oneKP |
| *VaplHEC3* | *Vanilla planifolia* | Orchidaceae (Vanilloideae) | VPTC006008 | Orchidstra |
| *OrsaHEC3* | *Oryza sativa* | Poaceae | LOC-Os08g01700 | Phytozome |
| *ZmHEC3* | *Zea mays* | Poaceae | GRMZM5G818776 | Phytozome |
| *ZmHEC3.2* | *Zea mays* | Poaceae | GRMZM5G802883 | Phytozome |
| **Basal Eudicots** | | | | |
| *PrhoHEC3* | *Papaver rhoeas* | Papaveraceae | IORZ-2009205 | oneKP |
| *PaseHEC3* | *Papaver setigerum* | Papaveraceae | STDO-2170060 | oneKP |
| **Core Eudicots** | | | | |
| *AtHEC3* | *Arabidopsis thaliana* | Brassicaceae | AT5G09750 | Phytozome |
| *AtIND* | *Arabidopsis thaliana* | Brassicaceae | AT4G00120 | Phytozome |
| *CaruHEC3* | *Capsella rubella* | Brassicaceae | Carubv10003900m | Phytozome |
| *CaruIND* | *Capsella rubella* | Brassicaceae | Carubv10033163m | Phytozome |
| *EusaIND* | *Eutrema salsugineum* | Brassicaceae | Thhalv10029526m | Phytozome |
| *RicoHEC3.1* | *Ricinus comunis* | Euphorbiaceae | XM_002517208 | Genbank |
| *MetrHEC3* | *Medicago truncatula* | Fabaceae | Medtr8g093500 | Phytozome |
| *ThecHEC3* | *Theobroma cacao* | Sterculiaceae | Thecc1EG001435t.1 | Phytozome |
| *ViviHEC3* | *Vitis vinifera* | Vitaceae | GSVIVT01026516001 | Phytozome |

Table S7: Accession numbers of *REPLUMLESS/POUND-FOOLISH* sequences used in the alignments and phylogenetic analyses. The Orchidaceae subfamilies are in parentheses.

| **Gene name** | **Species** | **Family** | **Accession number** | **Database** |
| --- | --- | --- | --- | --- |
| **Gymnosperms** | | | | |
| FataRPL | Falcatifolium taxoides | Podocarpaceae | ROWR-2007696 | oneKP |
| *MiteRPL* | *Microcachrys tetragona* | Podocarpaceae | MHGD-2076247 | oneKP |
| *NanaRPL* | *Nageia nagi* | Podocarpaceae | UUJS-2009138 | oneKP |
| **Basal Angiosperms** | | | | |
| *AsruPNF* | *Ascarina rubricaulis* | Chloranthaceae | WZFE-2196459 | oneKP |
| *GokePNF* | *Gomortega keule* | Gomortegaceae | MAQO-2119564 | oneKP |
| **Monocots** | | | | |
| *ApwaBEL1* | *Apostasia wallichii* | Orchidaceae (Apostasioideae) | AUTC015904 | Orchidstra |
| *NezoBEL1* | *Neuwiedia zollingeri* | Orchidaceae (Apostasioideae) | NZTC016779 | Orchidstra |
| *CyfoBEL1* | *Cypripedium formosanum* | Orchidaceae (Cypripedioideae) | CFTC008502 | Orchidstra |
| *CyenBEL1* | *Cymbidium ensifolium* | Orchidaceae (Epidendroideae) | CETC004797 | Orchidstra |
| *CysiBEL1* | *Cymbidium sinense* | Orchidaceae (Epidendroideae) | CSTC027834 | Orchidstra |
| *DenoBEL1* | *Dendrobium nobile* | Orchidaceae (Epidendroideae) | DNTC013235 | Orchidstra |
| *ErpuBEL1* | *Erycina pusilla* | Orchidaceae (Epidendroideae) | EPTC014626 | Orchidstra |
| *GaelBEL1* | *Gastrodia elata* | Orchidaceae (Epidendroideae) | GETC016657 | Orchidstra |
| *OngrBEL1* | *Oncidium gower ramsey* | Orchidaceae (Epidendroideae) | OGTC022811 | Orchidstra |
| *PhapBEL1* | *Phalaenopsis aphrodite* | Orchidaceae (Epidendroideae) | PATC124615 | Orchidstra |
| *PhbeBEL1* | *Phalaenopsis bellina* | Orchidaceae (Epidendroideae) | PBTC003626 | Orchidstra |
| *PheqBEL1* | *Phalaenopsis equestris* | Orchidaceae (Epidendroideae) | PETC019426 | Orchidstra |
| *PhluBEL1* | *Phalaenopsis lueddemanniana* | Orchidaceae (Epidendroideae) | PLTC003758 | Orchidstra |
| *PhmoBEL1* | *Phalaenopsis modesta* | Orchidaceae (Epidendroideae) | PMTC006233 | Orchidstra |
| *PhscBEL1* | *Phalaenopsis schilleriana* | Orchidaceae (Epidendroideae) | PSTC047228 | Orchidstra |
| *GopuPNF* | *Goodyera pubescens* | Orchidaceae (Orchidoideae) | VTUS_2003275 | oneKP |
| *HadiPNF* | *Haemaria discolor* | Orchidaceae (Orchidoideae) | LELS-2007092 | oneKP |
| *PlclBEL1* | *Platanthera clavellata* | Orchidaceae (Orchidoideae) | MTHW-2050883 | oneKP |
| *VaplBEL1* | *Vanilla planifolia* | Orchidaceae (Vanilloideae) | THDM-2005282 | oneKP |
| *OrsaRPL2* | *Oryza sativa* | Poaceae | LOC-Os05g38120 | Phytozome |
| **Basal Eudicots** | | | | |
| *AepuPNF* | *Aetoxicon punctatum* | Aetoxicaceae | QUTB-2017244 | oneKP |
| *BebePNF* | *Berberidopsis beckleri* | Flacourtiaceae | HAEU-2055923 | oneKP |
| *BebeRPL* | *Berberidopsis beckleri* | Flacourtiaceae | HAEU-2011390 | oneKP |
| **Core Eudicots** | | | | |
| *AlyrPNF* | *Arabidopsis lyrata* | Brassicaceae | AL4G22210.t1 | Phytozome |
| *AlyrRPL* | *Arabidopsis lyrata* | Brassicaceae | AL6G11240.t1 | Phytozome |
| *ATPNF* | *Arabidopsis thaliana* | Brassicaceae | At2g27990 | Phytozome |
| *ATRPL* | *Arabidopsis thaliana* | Brassicaceae | At5g02030 | Genbank |
| *CaruPNF* | *Capsella rubella* | Brassicaceae | Carubv10024860m | Phytozome |
| *CaruRPL* | *Capsella rubella* | Brassicaceae | Carubv10000448m | Phytozome |
| *GlymPNF* | *Glycine max* | Fabaceae | Glyma.13G324200.1 | Phytozome |
| *GlymRPL2* | *Glycine max* | Fabaceae | Glyma.01G104200.1 | Phytozome |
| *GlymRPL3* | *Glycine max* | Fabaceae | Glyma.18G189700.1 | Phytozome |
| *ThecPNF* | *Theobroma cacao* | Sterculiaceae | Thecc1EG042098t1 | Phytozome |
| *ThecRPL* | *Theobroma cacao* | Sterculiaceae | Thecc1EG021834t1 | Phytozome |
| *ViviPNF* | *Vitis vinifera* | Vitaceae | GSVIVG0102522001 | Phytozome |
| *ViviRPL* | *Vitis vinifera* | Vitaceae | GSVIVG01034073001 | Phytozome |

## Supplementary reference

Bustin, S.A., Benes, V., Garson, J.A., Hellemans, J., Huggett, J., Kubista, M., Mueller, R., Nolan, T., Pfaffl, M.W., Shipley, G.L., Vandesompele, J., and Wittwer, C.T. (2009). The MIQE guidelines: minimum information for publication of quantitative real-time PCR experiments. *Clin Chem* 55**,** 611-622.
